# Lanthanide‐Controlled Protein Switches: Development and *In Vitro* and *In Vivo* Applications

**DOI:** 10.1002/anie.202411584

**Published:** 2025-02-05

**Authors:** Zhong Guo, Oleh Smutok, Chantal Ronacher, Raquel Aguiar Rocha, Patricia Walden, Sergey Mureev, Zhenling Cui, Evgeny Katz, Colin Scott, Kirill Alexandrov

**Affiliations:** ^1^ ARC Centre of Excellence in Synthetic Biology Australia; ^2^ Centre for Agriculture and the Bioeconomy Queensland University of Technology Brisbane QLD 4001 Australia; ^3^ School of Biology and Environmental Science Queensland University of Technology Brisbane QLD 4001 Australia; ^4^ Department of Chemistry and Biomolecular Science Clarkson University 8 Clarkson Ave. Potsdam NY 13699 USA; ^5^ School of Engineering Sciences in Chemistry Biotechnology and Health KTH Royal Institute of Technology 100 44 Stockholm Sweden; ^6^ CSIRO Advanced Engineering Biology Future Science Platform Black Mountain Research & Innovation Park Clunies Ross Road Canberra ACT 2601 Australia

**Keywords:** protein biosensors, protein allostery, rare earth elements, lanthanides, protein engineering, synthetic biology

## Abstract

Lanthanides, which are part of the rare earth elements group have numerous applications in electronics, medicine and energy storage. However, our ability to extract them is not meeting the rapidly increasing demand. The discovery of the bacterial periplasmic lanthanide‐binding protein lanmodulin spurred significant interest in developing biotechnological routes for lanthanide detection and extraction. Here we report the construction of β‐lactamase‐lanmodulin chimeras that function as lanthanide‐controlled enzymatic switches. Optimized switches demonstrated dynamic ranges approaching 3000‐fold and could accurately quantify lanthanide ions in simple colorimetric or electrochemical assays. *E.coli* cells expressing such chimeras grow on β‐lactam antibiotics only in the presence of lanthanide ions. The developed lanthanide‐controlled protein switches represent a novel platform for engineering metal‐binding proteins for biosensing and microbial engineering.

## Graphical abstract

Engineering artificial allosteric protein switches controlled by rare earth elements allows the development of assays, construction of bioelectronic devices, and REE‐controlled microorganisms.

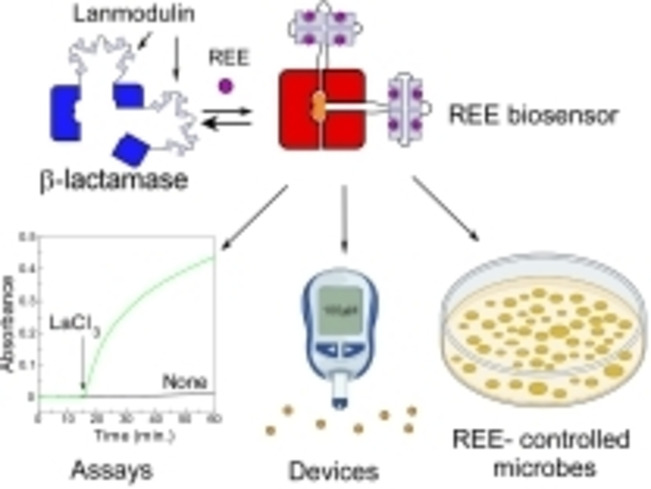

## Introduction

The demand for rare earth elements, including lanthanides (Lns), constantly increases, and their supply has been affected by geopolitical tensions^[1]^. These elements have diverse applications in electronics, medicine, energy storage, and clean energy generation[^2^, ^3^].Lns are chemically similar to one another and are generally found as insoluble phosphates, silicates, carbonates, and oxides. Because of this, methods that can extract and separate specific Lns are costly and laborious and often produce Ln‐contaminated waste. Therefore, new approaches for supplying Lns are needed.

Until the discovery of Ln‐dependent methylotrophic bacteria in 2005, Lns were not considered to have biological relevance.[^4^, ^5^] In 2011, it was shown that this Ln‐dependency was due to the role of a novel periplasmic, pyrroloquinoline quinone (PQQ)‐dependent methanol dehydrogenase (XoxF), that oxidises methanol to produce formaldehyde in a critical step for methylotrophy.^[6]^ Since this discovery, Lns have been shown to form the catalytic centre of a family of related aldehyde‐forming, PQQ‐dependent alcohol dehydrogenases, fulfilling the role of Lewis acid, as does Ca^2+^ in the PQQ‐dependent MxaF‐family of enzymes.[^7^, ^8^]

More recently, a periplasmic protein with high selectivity and avidity for Lns, lanmodulin (LanM), was identified.^[9]^ LanM binds Lns in periplasm and is thought to trigger the expression of a lanthanide‐induced regulon (sometimes termed the lanthanome). Structural studies have revealed that LanM contains four EF‐hands, similar to those found in the calcium‐binding sites of calmodulin, but with a single proline residue that substantially decreases its affinity for calcium compared with EF‐hands lacking such residue.^[10]^ The binding of Lns to EF‐hands is cooperative, leading to conformational changes in LanM and engendering low *K*_D_ for these cations.

LanM was adapted for use as an Ln biosensor or chelator for the bioseparation of Lns.[^11^, ^12^, ^13^] LanM‐functionalised beads were shown to effectively enrich Lns from a solution of mixed cations in a packed‐bed reactor, binding to the column and then released using low pH or with a chelator such as citrate. More recently, an orthologue of the well‐studied *Methylorubrum extorquens* LanM isolated from *Hansschlegelia quercus* was shown to possess a substantially lower *K*_D_ for NdIII than for DyIII and was used in selective separation of the two Lns from a mixture.^[14]^

To date, three approaches have been explored to utilise LanM as a biosensor. First, LanM was fused with FRET‐forming fluorescent proteins to take advantage of its large conformational changes upon metal binding. This approach has produced biosensors with picomolar *K*_D_ for Lns.^[12]^ This FRET‐based biosensor was also re‐engineered to detect manganese.^[15]^ The second approach exploited the natural luminescence of some lanthanides (e.g., TbIII) in which a photosensitiser (a tryptophan residue) was included in the EF‐hand in proximity to the bound Ln to promote luminescence resonance transfer.^[16]^ Finally, the LanTERN sensor is a repurposed GCaMP sensor in which the calcium‐binding EF‐hands were replaced with LanM‐inspired EF hands that bind lanthanides with higher selectivity than calcium.^[17]^

Although LanM possesses remarkable selectivity for Lns, it does not have enzymatic activity by itself. Therefore, in the present study, we sought to expand the utility of LanM by integrating it into a higher‐order synthetic protein system that would convert Ln binding into enzymatic and biological activity. This, for instance, can be achieved by constructing artificial chimeric allosteric enzymes.[^18^, ^19^] Among possible enzymatic reporters β‐lactamase (BLA) family members have been used extensively because of their small size, high catalytic activity, large selection of reporter substrates, and the ability to endow Gram‐negative bacteria with β‐lactam antibiotic resistance.^[20]^ Pioneering studies demonstrated the construction of a chimera between TEM‐1 β‐lactamase and a solute‐binding protein that displayed a >600‐fold increase in activity in the presence of maltose.^[21]^ Such a large dynamic range was achieved through extensive mutational optimisation of less potent chimeras identified in the initial antibiotic resistance screen. Other groups were also able to construct chimeric switches based on TEM‐1 but reported more modest dynamic ranges.^[22]^

We previously demonstrated that the dynamic range and catalytic activity of artificial chimeric proteins are anticorrelated, presumably because of a thermodynamic trade‐off that prevents simultaneous optimisation of protein switches for dynamic range, catalytic activity and response rate.^[23]^ However, we later proposed a solution to this problem in which the introduction of two identical receptor domains into a protein chimera resulted in the emergence of switching with large dynamic ranges, rapid activation and high catalytic activity.^[24]^ In the this study, we used these engineering principles to construct chimeras of LanM and TEM‐1 β‐lactamase that function as Ln biosensors. We demonstrate that these biosensors can be used to quantify Lns in solution using colorimetric and electrochemical methods. We also show that *E. coli* strains expressing β‐lactamase‐lanmodulin chimera display Ln‐dependent growth on ampicillin.

## Results and Discussion

### Construction and Functional Analysis of a Lanmodulin‐β‐lactamase Chimera Library

**Figure 1:**
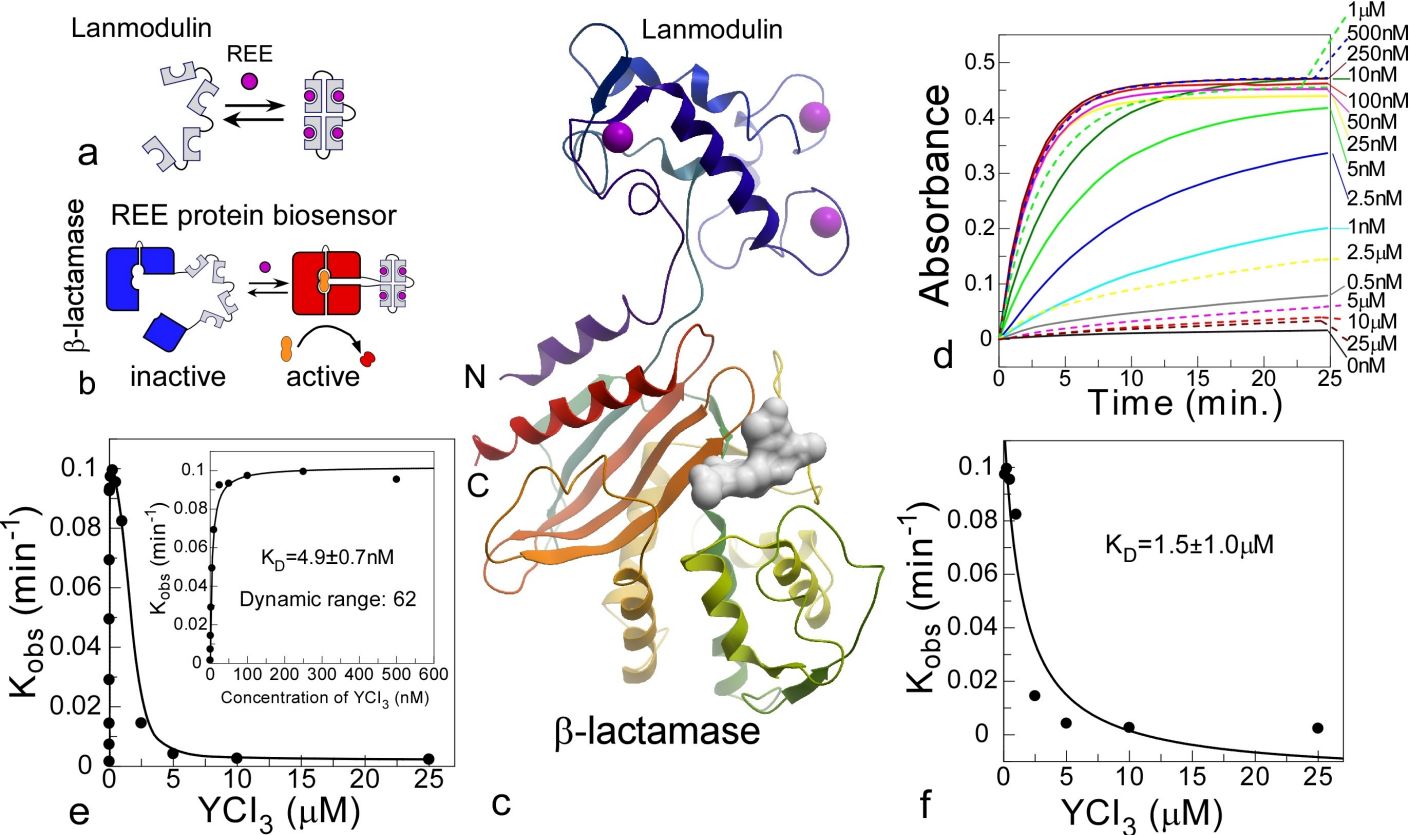
*Construction and characterization of Ln biosensors based on lanmodulin‐β‐lactamase (LanM‐BLA) chimeras*. (a) Schematic representation of lanmodulin and Ln‐induced changes in its structure. (b) A schematic of LanM‐BLA chimera and putative mechanism of its activation by Lns. (c) A model of LanM‐41‐BLA chimera based on the PDB structures 6mi5 and 3gmw displayed in ribbon representation. The molecule is colored spectrally from N− to C− termini and the ions are displayed as magenta spheres. The active site of BLA is marked by an inhibitor imported from PDB:6c79 and displayed as white molecular surface . (d) Time‐resolved changes in the absorption of β‐lactam UW154 following its hydrolysis by 10 nM LanM‐41‐BLA chimera in the presence of increasing concentrations of YCl_3_. (e) A plot of k_obs_ values extracted from the experiments shown in (d) plotted against the concentration of YCl_3_. The inset shows the fit of the first phase to a quadratic equation, while the panel (f) shows the fit of the second phase of the reaction to an apparent *K*_D_ of 1.5 μM.

To create a protein system in which LanM controls the activity of an enzymatic reporter, we examined the crystal structure of TEM‐1 β‐lactamase (PDB:3gmw) to identify surface loops where LanM insertion was less likely to perturb the overall fold of TEM‐1 and, ligand‐induced conformation could propagate to the enzyme's active site (Figure 1).

Based on this analysis we designed and synthesized ten chimeric genes and recombinantly produced the corresponding proteins in *E. coli* (Supporting Information Table 2). The activities of the purified proteins were assessed in the presence or absence of 0.5 μM yttrium chloride and the chromogenic β‐lactamase substrate UW154 (Figure S1). Most of the chimeras displayed ion‐dependent activation, albeit with different dynamic ranges, maximal catalytic activities, and activation rates (time required to reach full activity) (Figure S1). The chimera with the highest catalytic rate in the activated state had LanM inserted at position 41 of TEM‐1 displayed a dynamic range of 62‐fold, and, was fully activated within 10 min of exposure to yttrium chloride (Figure 1c–e and Figure S1). Chimeras with insertion at positions 228, 240, and 253 also displayed dynamic ranges of >10‐fold but had lower maximal catalytic rates (Figure S1 h, i, j).

Interestingly, this chimera displayed a nonlinear response to yttrium concentration. The maximal catalytic activity of this construct was achieved at 100 nM of Y^3+^ and further increases in its concentration resulted in activity decrease to almost ground level (Figure 1e). The loss of activity at high Y^3+^ was not a consequence of BLA activity inhibition by yttrium, as it had little effect on BLA activity at concentrations of up to 25 μM (Figure S1 k). As the Y^3+^‐dependent activation and inactivation phases were well separated, we estimated the *K*_D_ values for each phase to be 5 nM and 1.5 μM, respectively. The activity change was not a consequence of the change in the chimera's oligomerization state as it eluted from a size exclusion column as a monomer in the absence and presence of Y^3+^ (Figure S2 a).The chimera with the second largest dynamic range (35‐fold) was LanM‐253G‐BLA, which also exhibited a biphasic activation‐inactivation behavior with binding equilibrium constants of 5.4 nM and 1.0 μM (Figure S2 b–d). The similar behavior of two structurally different chimeras suggests that they reflect binding of the yttrium ion to LanM and the propagation of the resulting conformation changes into BLA. The second binding phase possibly reflects the interaction of the ion with the low affinity EF hand, which induces a conformation change that reverses BLA activation. Most likely this relates to the previously characterised multiple conformational transitions of LanM mediated by the differences in ligand affinities of individual EF‐hands for Lns.^[16]^

**Figure 2:**
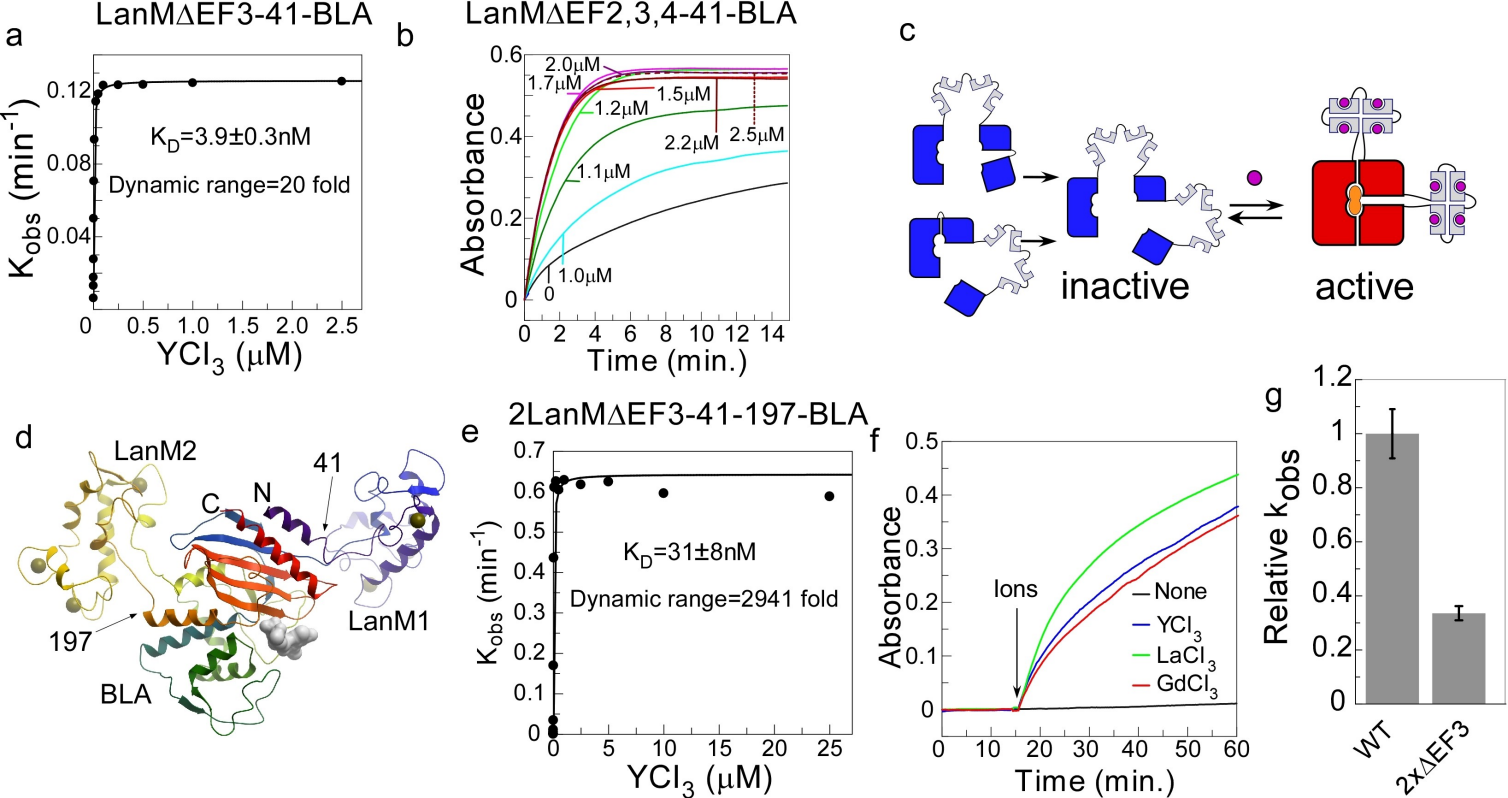
*Construction of biosensor variants with improved performance*. (a) A plot of k_obs_ values obtained by titrating 10 nM of the LanMΔEF3‐4141‐BLA variant with increasing concentrations of YCl_3_. The fit to a quadratic equation resulted in a *K*_D_ value of ca. 4 nM. (b) Absorption traces obtained using 25 nM of the biosensor with a single intact EF‐hand 1 (LanM‐ΔEF2,3,4‐41‐BLA) in the presence of increasing concentrations of YCl_3_. (c) A schematic representation of the approach for constructing the YES‐gate biosensor through a combination of chimeras with LanM inserted at different positions of β‐lactamase. (d) A model of chimeric β‐lactamase with lanmodulin insertions at positions 41 and 197 (marked by arrows). The structure is spectrally coloured from marked N‐ to C‐ termini. (e) A plot of k_obs_ values obtained by titrating 25 nM of 2LanMΔEF3‐41‐197‐BLA variant with increasing concentrations of YCl_3._ The fit to a quadratic equation resulted in a *K*_D_ value of ca. 31 nM. (f) Time‐resolved change in absorption of a solution of 50 μM UW154 and 25 nM of 2LanMΔEF3‐41‐197‐BLA upon addition of yttrium, lanthanum and gadolinium chlorides to a final concentration of 1 μM. (g) Bar chart comparing the relative catalytic activity of 10 nM of wild‐type TEM‐1 BLA and the 2LanMΔEF3‐41‐197‐BLA variant activated by 1 μM of YCl_3._ The error bars denote positive and negative boundaries of the standard error of mean.

**Construction of LanM‐41‐BLA chimeras with reduced number of EF‐hands**. While interesting from the perspective of LanM function, the biphasic behavior of the biosensor creates potential problems for its application, as two significantly different concentrations of Lns will result in the same signal intensity. Therefore, we attempted to simplify the chimera's behavior and identify the site responsible for the biosensor's inhibition at high concentrations by iterative ablation of individual EF‐hands. We thus systematically mutated all EF‐hands in the gene encoding the LanM‐41‐BLA chimera, substituting all metal‐coordinating aspartate side chains with serine residues and re‐analyzing the activity of the resulting chimeras in response to yttrium ions (Figure 2 a,b, Figure S3, Supporting Information Table 2). All variants retained their responsiveness to yttrium ions and displayed *K*_D_ values below 10 nM suggesting that activation of the reporter was not dependent on a single EF‐hand^[9]^. This is consistent with the notion that EF‐hands act cooperatively leading to a global structural rearrangement of LanM. To confirm the obtained values we performed a reverse titration where La^3+^:LanMEFΔ3 −41‐BLA complex was titrated with EDTA, resulting in a dose‐dependent signal decrease in the signal intensity. The fit of the data to a competitive model resulted in a *K*_D_=9 nM confirming that the direct titrations provided an accurate estimation of the affinity (Figure S3 m). Remarkably, mutations of the EF3‐hand (LanMEFΔ3‐41‐BLA) resulted in a biosensor that more closely resembles a classical OFF‐to‐ON switch at ion concentrations up to 2.5 μM, albeit some inhibition was observed at a concentration of YCl_3_ above 5 mM. A similar effect was observed in the case of LaCl_3_, and it was more pronounced with GdCl_3_, although in this case inhibition could be attributed to its direct effect on BLA activity (Figure S3 g, h, k, l and 1 k, l, m).

As expected, the biosensor was not activated by other metal ions such as Fe^3+^, Al^3+^, Ca^2+^ and exhibited minor activation at 5 mM of Mn^2+^ (Figure S4). This is consistent with an earlier report in which mutational modification of the lanmodulin enhanced its specificity for Mn.^[15]^ The observation that all EF‐hand variants were still able to communicate with the BLA reporter is of both fundamental and practical importance, as understanding and engineering of LanM's ligand specificity is complicated by the highly cooperative nature of EF‐hand interactions in the context of the protein.[^9^, ^25^] LanM‐BLA chimeras with reduced numbers of EF‐hands may allow us to elucidate the contribution of individual binding sites to the behavior of the overall system and ultimately LanM variants with altered ligand specificity. To this end we set out to identify the minimal number of EF‐hands that allow the chimera to retain Ln‐mediated activity. We constructed LanM‐41‐BLA variants by combining mutations developed earlier, resulting in ΔEF1,3; ΔEF2,3 and ΔEF3,4 variants. While the ΔEF2,3 chimera showed reduced catalytic activity and low dynamic range, the ΔEF1,3 and ΔEF3,4 chimeras retained high catalytic activity and a large dynamic range (42 and 128‐fold respectively) (Figure S5, Supporting Information Table 1). Interestingly, both exhibited a very steep response to the ligand ion, which is possibly a result of of cooperativity collapse among the EF‐hands (Figure S5).^[25]^ Guided by these results we constructed a LanM‐41‐BLA chimera containing only one functional EF‐hand (EF1) but retained ion responsiveness and relatively high affinity (*K*_D_=35 nM), albeit with a lower dynamic range and maximal catalytic activity than variants with two or more EF‐hands (Figure 2b, Figure S5 f and Supporting Information Table 1). This chimera is a valuable tool for future EF‐hand engineering, as it provides a simple and direct functional readout that enables rapid analysis of large combinatorial variant libraries of EF‐hand mutations.

#### Development of YES‐gate Rare Earth Biosensors

**Figure 3:**
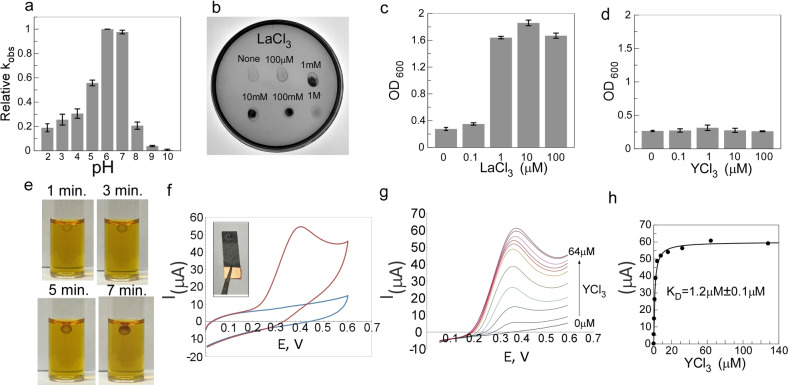
*In vitro* and *in vivo* analysis of the 2LanMΔEF3‐41‐197‐BLA chimera. (a) A plot of the pH dependence of 2LanMΔEF3‐41‐197‐BLA biosensor activity. (b) An agar plate containing ampicillin and chloramphenicol antibiotics seeded with *E. coli* strain DH5α expressing 2LanMΔEF3‐41‐197‐BLA chimera. After bacterial plating 2 μl of LaCl_3_ with indicated concentration were spotted at the indicated positions and bacterial growth was allowed to develop overnight. (c) A density plot of *E. coli* DH5α suspension culture of 2LanMΔEF3‐41‐197‐BLA in LB liquid medium in the presence of the indicated concentrations of LaCl_3_. (d) As in (c) but in the presence of YCl_3_. (e) Visualization of time‐dependent UW154 hydrolysis by 2LanMΔEF3‐41‐197‐BLA chimera entrapped in chitosan/alginate composite hydrogel bead in the presence of 100 μM YCl_3_. The color change of the bead indicates accumulation of the hydrolysed UW154 product. (f) Typical cyclic voltammograms of the output of 2LanMΔEF3‐41‐197‐BLA‐chitosan/alginate composite bioelectrode in the presence and absence of lanthanide ions. Blue curve: 300 μM UW154, 100 μM YCl_3_ scanned at the rate of 50 mV/s vs. Ag/AgCl/3 M reference electrode at room temperature. The red curve represents the same reaction conditions scanned after a 10‐minute incubation. (g) Typical linear sweep voltammograms of 2LanMΔEF3‐41‐197‐BLA‐chitosan/alginate composite bioelectrode in the presence of 300 μM UW154 and increasing YCl_3_ concentrations. The inset shows carbon paper electrode functionalized with the biosensor‐containing gel used in these experiments. (f) Fit of the titration data shown in (g) to a quadratic equation leading to an apparent *K*_D_ value of 1.2 μM.

Protein biosensors typically require extensive mutational optimization to achieve the desired balance of dynamic range, sensitivity, and catalytic activity.[^26^, ^27^] This is not always straightforward as biosensor performance parameters are thermodynamically coupled.^[23]^ We demonstrated previously that some of the inherent thermodynamic trade‐offs can be overcome by duplicating receptor domains of the chimeras, thereby increasing the dynamic range without a large penalty on the catalytic activity or the activation rate.[^23^, ^24^] After resolving the problem of biosensor inhibition at high concentrations of Lns by disrupting EF3 hand, we constructed a chimera of β‐lactamase with ΔEF3 variant of lanmodulin incorporated at positions 41 and 197 (Figure 2d). The resulting chimera was produced recombinantly in good yields and displayed an exceptional dynamic range of nearly 3000‐fold, while retaining ca. 34 % activity of the wild‐type enzyme (Figure 2e, f). This YES‐gate biosensor had a pH optimum of 6.0–7.0, but some activity could be detected at pH 2.0 (Figure 3a). We were able to further improve the maximal catalytic activity to *ca*. 58 % of the wild‐type enzyme by introducing known stabilizing amino acid substitutions into this chimera (Figure S6 a).^[24]^ We were also able to construct YES gate switches using other EF‐hand mutants among which 2LanMΔEF3,4‐41‐197‐BLA displayed the largest dynamic range of 1938 folds (Figure S6 b–f, Supporting Information Table 1). In these variants, we observed a more pronounced inhibition at high metal ion concentration (Figure S6 b–f). This suggests that wild‐type BLA may not be an ideal control for the non‐EF hand‐mediated effects of lanthanides on the developed biosensors. We hypothesized that the insertion of LanM may have altered the susceptibility of BLA to metal ions. To test that we constructed a LanMΔEF1,2,3,4‐BLA chimera that displayed approximately 7.5 % of wild‐type activity (Supporting Information Table 1). As expected, the exposure of this chimera to lanthanide salts did not result in activity increase at nM concentrations but showed activity inhibition in the μM range (Figure S5 e). This confirms that the biosensor's inhibition at high concentrations of lanthanide salts is likely to be unrelated to LanM function and possibly reflects interactions of Ln with more exposed hydrophobic core of the chimeric protein. To further ascertain that mutated EF hands were not able to bind Lns ions we constructed a LanM‐PQQ‐glucose dehydrogenase chimera that acted as a Lns biosensor with the output orthogonal to that of LanM‐BLA (Figure S5 g, h). Using this biosensor we were able to demonstrate that LanMΔEF1,2,3,4‐41‐BLA chimera retained no detectable Lns binding activity (Figure S5 i).

#### Construction of E. coli Strains Expressing LanM‐BLA Biosensors

The ability of β‐lactamase to endow Gram‐negative bacteria with resistance to β‐lactam antibiotics offers an exciting opportunity for the construction of bacterial cells “addicted” to rare earth metals. To test this idea, we constructed a recombinant *E. coli* strain that produced 2LanM‐BLA chimera in the periplasm under the control of a constitutive promoter. The resulting strain was able to grow on both liquid and solid medium supplemented with ampicillin only in the presence of micromolar concentrations of LaCl_3_ and GdCl_3_ (Figure 3, Figure S7). We also were able to monitor the Lns‐dependent β‐lactamase activity of LanM biosensors using transformed *E.coli* cells suspension or even culture supernatants (Figure S7 i, 8 a–c).

Interestingly, although the strain grew well in the presence of gadolinium and lanthanum, yttrium was not able to support bacterial growth on solid or in a liquid medium (Figure 3 d, Figure S7 e, f, g). However, we could detect yttrium‐dependent β‐lactamase activity intact cells (Figure S7 i). Given that yttrium chloride is not toxic to *E.coli*, the mechanism underlying this difference remains unclear.

#### Construction and Testing of Bioelectrodes Based on LanM‐BLA Chimera

One of the significant advantages of β‐lactamase reporter is the abundance of methods for its activity detection that stems from a century‐long warfare between medicinal chemists and pathogenic bacteria. As a result, a plethora of fluorescent, colorimetric, luminescent, turbidimetric and electrochemical reporters have been developed.^[20]^ It was previously shown by us and others that the hydrolysis of nitrocefin could be monitored electrochemically.[^24^, ^28^] We wanted to explore whether the activity of the developed lanthanide biosensors could be monitored electrochemically and whether the β‐lactamase substrate UW154 used in this study could be a suitable electrochemical reporter. As the previously used alginate gel was not suitable for the construction of the electrodes due to its dependency on trivalent ions, we tested different gel‐forming materials and identified the mixture of chitosan/alginate as an optimal medium allowing for the diffusion of the biosensor's ligands and β‐lactamase substrates. As can be seen in Figure 3e 2LanMEFΔ3‐41‐197‐BLA gel‐embedded biosensors could hydrolyze UW154, resulting in progressive accumulation of the colored product in the gel. To construct a sensory electrode, we polymerized a biosensor‐containing gel system on the surface of a carbon‐paper electrode (Supplementary Figures 9–10). When this electrode was exposed to a solution of yttrium chloride and UW154, the cyclic voltammograms revealed a large increase in the current at 0.4 V (vs. Ag/AgCl) indicative of β‐lactam hydrolysis (Figure 3f). Importantly, the bioelectrode could be stored in a dehydrated form with minimal loss of activity upon rehydration (Figure S10 d). Titration of the electrode with Ln ions resulted in a dose‐dependent and saturable increase in current that was used to quantify the yttrium and lanthanum ions in solution (Figure 3 g, h, Figure S11). This provides an opportunity for the production of low‐cost analytical systems for the rapid quantification of rare earth ions in industrial and environmental samples.

## Discussion

Here we present a new class of rare earth biosensors based on a lanmodulin‐β‐lactamase chimeras. Unlike previous biosensor designs where the binding of ions modulated the distance between FRET‐forming fluorescent proteins, altering the protonation state of a fluorophore, or luminescence of the metal itself, the current design is based on allosteric modulation of enzymatic activity of β‐lactamase.[^12^, ^15^] Due to the nature of fluorescent protein‐based FRET or intensitometeric biosensors tend to have limited dynamic range.[^26^, ^29^, ^30^]

In contrast, biosensors with catalytic reporters can provide much larger dynamic ranges due to enzymatic signal amplification. Catalytic biosensors also provide a larger selection of outputs that can be tailored to different applications. Therefore, we chose to design a biosensor based on highly catalytically active β‐lactamase that has a range of reporter substrates.^[20]^ Using a semi‐rational domain insertion strategy, we produced a focused library of TEM‐1 β‐lactamase:lanmodulin chimeras of which *ca*. 40 % were switches with useful performance parameters. However, we observed a nonlinear biosensor response to the lanthanide ions, which limited the utility of our initial designs. The initial increase in activity with the apparent low nM *K*_D_ was followed by activity inhibition with an apparent low μM *K*_D_. Given that LanM has four metal binding sites (EF‐hands) with different affinities, this is not surprising *per se* as it is easy to imagine that conformational changes of individual sites could propagate differently onto the reporter domain. Additionally, the NMR structure of LanM revealed that the EF‐hand 4 was not occupied by a cation, suggesting that it may have a lower affinity for the ligand than the other three EF‐hands.^[10]^ Interestingly, mutational analysis of the chimera revealed that EF4 ablation had no detectable effect on the biosensor's biphasic response, whereas EF3‐hand mutation largely removed it. Therefore, it appears likely that in the context of a chimera EF3 has μM affinity for lanthanides and induces an additional conformational change in LanM‐BLA chimera, leading to its deactivation.

By combining single EF‐hand mutations, we constructed a biosensor containing only one functional EF‐hand 1. The ability to monitor metal binding to a single EF‐hand through an enzymatic output presents an exciting prospect for EF‐hand specificity engineering. We used the developed EFΔ3 mutant to construct an advanced version of REE biosensors by incorporating it into two different positions in β‐lactamase, effectively creating an intramolecular YES‐gate switch with much more efficient OFF state compared to single‐insertion chimeras.^[24]^ The optimized variant demonstrated a nearly 3000‐fold dynamic range while retaining 30 %–60 % activity of the wild‐type enzyme. Such large dynamic ranges have not been described for naturally occurring equilibrium systems thereby showcasing the power of rational protein switch design.

When expressed in *E. coli*, the developed β‐lactamase chimeras with single and double LanM EFΔ3 insertions produced strains that could grow on ampicillin only in the presence of μM concentrations of lanthanide ions. Switches with such large dynamic ranges are likely to be very useful for *in vivo* evolution experiments as antibiotic resistance cannot be gained by switch overexpression which is a common microbial adaptation that subverts *in vivo* directed evolution experiments. This gives us a powerful LanM engineering tool that combines antibiotic selection and high‐throughput enzymatic analysis of the individual clones.The latter notion is supported by our observations that activity of LanM‐based biosensors could be monitored using suspension of biosensor‐expressing *E.coli* cells. Our experiments also highlight the potential utility of the developed biosensors for the construction of microbial strains capable of extracting lanthanides from various sources.

Finally, we tested the suitability of the developed YES gate switches for the construction of bioelectrodes for quantifying lanthanides. We trapped the enzyme in a gel composed of alginate and chitosan and demonstrated that the biosensors retained their activities under these conditions. Deposition of such gel on the surface of the electrodes allowed us to use cyclic voltammetry to monitor hydrolysis of β‐lactam substrate stimulated by the presence of lanthanides. Our results show the feasibility of the construction of inexpensive lanthanide‐detecting electrodes that coupled with a platform for engineering and evolution of the principal biosensor opens numerous opportunities in lanthanide mining and recycling.

## Conflict of Interests

The authors declare no conflict of interest.

1

## Supporting information

As a service to our authors and readers, this journal provides supporting information supplied by the authors. Such materials are peer reviewed and may be re‐organized for online delivery, but are not copy‐edited or typeset. Technical support issues arising from supporting information (other than missing files) should be addressed to the authors.

Supporting Information

## Data Availability

The data that support the findings of this study are available in the supplementary material of this article.
