# Supplementary material for: Lanthanide‐Controlled Protein Switches: Development and In Vitro and In Vivo Applications
Source: Angew Chem Int Ed Engl. 2025 Feb 5;64(9):e202411584. doi: 10.1002/anie.202411584 (PMC11848957; doi:10.1002/anie.202411584)
Supplement: Supplementary file 1 — Supporting Information [file ANIE-64-e202411584-s001.pdf]

Angewandte Chemie
Eine Zeitschrift der Gesellschaft Deutscher Chemiker

Supporting Information

# Lanthanide-Controlled Protein Switches: Development and *In Vitro* and *In Vivo* Applications

*Z. Guo, O. Smutok, C. Ronacher, R. Aguiar Rocha, P. Walden, S. Mureev, Z. Cui, E. Katz, C. Scott, K. Alexandrov**

**Supplementary Information**

# Lanthanide-controlled protein switches: development and in vitro and in vivo applications

Zhong Guo, Oleh Smutok, Chantal Ronacher, Raquel Aguiar Rocha, Patricia Walden, Sergey Mureev, Zhenling Cui, Evgeny Katz, Colin Scott and Kirill Alexandrov

## Materials:

Lanthanide salts were purchased from Sigma-Aldrich. UW154 was synthesized by o2 Discovery Inc. according to the previously published protocol[28].

## Methods:

### Molecular cloning, expression, and purification of recombinant proteins

The open reading frames of the constructs listed in Supplementary Table 2 were synthesized commercially (Gene Universal) and cloned into a kanamycin-resistant pET-28a(+) vector or , for *in vivo* experiments into modified pACYCDuet-1 where T7 promotor was replaced by the constitutive AmpR promotor[23]. Competent *E. coli* BL21(DE3) cells (New England Biolabs) were transformed with the resulting expression vectors and grown in LB broth containing 50 µg/mL kanamycin (Everest Inc, Australia) with shaking at 37 °C. Protein expression was induced by adding 0.3 mM IPTG (Sigma, USA) and the cultures were incubated overnight at 18 °C. Cells were harvested at 4,000 rpm for 10 min, and the cell pellet was lysed in buffer containing 50 mM $NaHPO_4$ (pH 8.0), 300 mM NaCl, 20 mM imidazole, 1 mM AEBSF and DNAse I (Sigma-Aldrich, USA) using the CFII cell disrupter at 27 kPsi (Constant Systems, UK). The). T lysate was cleared by centrifugation and supernatant applied onto on the Ni-NTA HisTrap FF crude column driven by ÄKTA Express Purifier (Cytiva) system in buffer containing 50 mM $NaHPO_4$ pH8.0 300 mM NaCl and 20 mM imidazole. The column was developed with a gradient of the same buffer containing 500 mM imidazole. TheT fractions containing the desired protein were dialyzed against 20 mM Tris-HCl (pH 7.2) and 100 mM NaCl and protein aliquots were frozen in liquid nitrogen and stored at -80 °C. Protein concentration was measured using NanoDrop OneOne C (Thermo Fisher Scientific).

### *Construction of E.coli strains expressing LanM-BLA chimeras*

LanM-BLa chimeras were cloned into a modified pColaDuet-1 vector where the T7 promoter was replaces with a tac promoter. Transformation and protein expression was performed in

NEBExpress® Competent *E. Coli* (New England Biolabs). A plasmid carrying kanamycin resistance gene and expressing cog3 protein instead of β-lactamase was included as the negative control. Protein expression in suspension culture was induced with 0.1 mM IPTG overnight at 25°C. Supernatant of $OD_{600}$ normalized cultures was harvested by centrifugation at 14, 000 rpm for 10 min. Kinetic assays were performed as described below, directly adding supernatant or suspension culture to the reaction mix and incubating with 500 nM $LaCl_3$ for 15 minutes prior to measurement.

**Spectrophotometric analysis of β-lactamase enzymatic activity**

EnzymaticE β-lactamase assays were performed in the assay buffer containing 20 mM Tris-HCl (pH 7.2), and 100 mM NaCl at 25°C by monitoring the increase in absorbance of UW154 at 520 nm using a Cary 60 UV-VIS absorbance spectrometer (Agilent) operated by Cary WinUV Software. The assay volume was kept at 1 mL1ml.

**Spectrophotometric analysis of PQQ-GDH enzymatic activity**

The PQQ-GDH-based chimeras were reconstituted with PQQ in 1:1.5 ratio. The GDH enzyme assay was performed as described previously[41]. Briefly, the 1.0 mL volume reactions containing 20 mM glucose, 0.6 mM phenazine methosulfate (PMS), 0.06 mM 2,6 dichlorophenylindophenol (DCPIP), 20 mM Tris-HCl, pH 7.2, 100 mM NaCl, 100 μM $CaCl_2$ and the appropriate enzyme were carried out in polystyrol cuvettes (SARSTEDT). The assays were performed at 25 °C by monitoring the decrease in absorbance of DCPIP at 600 nM using a Cary 60 UV-VIS absorbance spectrometer.

**Quantification of biosensor performance parameters and data plotting**

To determine the affinity of the biosensors for their targets the linear phase of the curves recorded at different concentrations of the ligand were fitted as a linear function to obtain $k_{obs}$. To obtain the $K_D$ for the interaction of biosensors with their ligand, the $k_{obs}$ data were plotted against the concentration of the ligand and the data were fitted to the explicit solution of the quadratic equation describing the *E + S <> ES* binding equilibrium, where $K_D$ is defined as:

$K_D = [E]*[S]/[EL]$. $[E_0]$ and $[L_0]$ refer to the total enzyme and ligand concentration (free and bound) in the cuvette. Under these conditions the absorbance (Obs) is described by:

$$Obs = K_{obs\,(min)} + (K_{obs\,(max)\,x} - K_{obs\,(min)})/_* (([E_0]+ [L_0]+K_d)/2- ([E_0]+ [L_0]+ K_d)^2/4 - [E_0]*[L_0])^{1/2} /[L_0]$$

$K_{obs}$ represents the measured rate, while $K_{obs\,(min)}$ and $K_{obs\,(max)}$ refer to the minimal and maximal rates observed, respectively. A least-squares fit of the data to equation 1 using the software package Grafit 5.04 (Erithacus software) was used to extract the $K_D$ value.

To determine the *dynamic range* of the individual switches ligand titration experiments were first performed to establish the $K_D$ value for the ligand. The assays were then repeated either at zero or at the saturating concentration of the ligand (typically 20-fold of the $K_D$ value) and absorbance trace was recorded. The linear phase of the trace was fitted to the linear function toto give the $k_{obs}$. The dynamic range was calculated by dividing the $k_{obs}$ of the background signal in the absence of the ligand by the $k_{obs}$ recorded at saturating ligands concentration.

To determine the rate of biosensor's response, the biosensors were incubated with Lns for the indicated period of time and assayed for enzymatic activity. The initial rates were determined, and the activation rate was calculated by fitting the data to a single exponential equation.

**Competitive titration of chimeric biosensors and the data analysis**

The competitive titration was performed using 1ml of a mixture of 25 nM25nM LanM∆EF3-41-BLA chimera with 25 nM25nM of $LaCl_3$ and titrating it with the increasing concentrations of EDTA in the assay buffer containing 20 mM Tris-HCl (pH 7.2), and 100 mM NaCl. The increase in absorbance of UW154 at 520nm at 25°C was monitoring monitored by using a Cary 60 UV. A plot of the $k_{obs}$ values obtained by the competitive titration. The data was fitted by GraphPad Prism 10.0.3 by using a One Site competitive model form the Fit Kit that uses the following equation:

logEC50=log(10^logKi*(1+HotNM/HotKdNM))

Where the parameters were defined as follows:

Y= (Top-Bottom)/(1+10^(X-LogEC50)) + Bottom

X: logmolar concentration of EDTA

HotNM: concentration of LanM∆EF3-41-BLA sensor

Y: $k_{obs}$

Top and Bottom: the highest and the lowest $k_{obs}$, respectively

Ki: dissociation constant of $LaCl_3$ and EDTA

HotKdNM: dissociation constant of $LaCl_3$ and LanM∆EF3-41-BLA sensor

**Gel filtration analysis of LanM-41G-BLA chimera**

The size exclusion chromatography was performed with 2 mg of LanM-41-BLA chimera on Superdex 75 16/60 gel filtration column equilibrated with 20 mM Tris pH7.2, 100 mM, 100mM

NaCl and the absence or presence of 1μM $LaCl_3$. The position of molecular weight markers used for calibration of the column were purchased from Sigma-Aldrich.

**pH tolerance analysis of the 2LanMEFΔ3-41-197-BLA chimera**

To test the pH tolerance of our biosensor we incubated 25nM 2LanMEFΔ3-41-197-BLA chimera in triplicates at different pH for 15 minutes followed by β-lactamase activity assay. The assay was carried out at 20mM Tris pH 2-10, 100 mM NaCl, 50μM UW154 and 500 nM $YCl_3$.

**Preparation of alginate-chitosan gel matrix containing LanM biosensor and its use for bio-electrode construction**

For the gel preparation, 15 μL of 2LanMEFΔ3-41-197-BLA chimera 25 μM stock solution was mixed with 0.3 mL of chitosan hydrogel (200 mg of medium molecular weight chitosan in 6 mL of 0.75 % v/v acetic acid). The enzyme-modified chitosan hydrogel (pH 5.8-6) was intensively mixed, centrifugated for 5 min. at 8000 rpm in 10 cm centrifugation vessel to remove oxygen bubbles and stored at +8$^{o}$C until use. To provide the electrode with the maximal biosensor loading, 15 μL of the matrix was applied to both sides of the carbon paper electrode. The matrix was subsequently overlayed by 15 μL of 1.7% alginate and the electrode was dried for 30-40 minutes at room temperature to ensure penetration of alginate into the chitosan layer and its crosslinking and stabilization. The formed enzyme-chitosan/alginate composite bioelectrodes were washed with 25 mM Tris-HCl buffer, pH 7.2 before use.

**Analysis of electrochemical properties of UW154 hydrolysis products**

To analyse the electrochemical behaviour of electroactive products of enzymatic hydrolysis of 300 μM UW154 a cyclic voltamperometry was performed using 100 μM $YCl_3$ as a target analyte. A high oxidation peak was observed at +0.38 V vs. Ag/AgCl/3M KCl which corresponds to the electrochemical oxidation of the electroactive product of enzymatic hydrolysis of UW154. However, after numerous experiments, it was estimated that this oxidation peak could be shifted to +0.365 V vs. Ag/AgCl/3 M KCl, especially in the case of usage of linear sweep voltammetry instead of cyclic voltamperometry. The reason for such variation remains unclear. As oxidation at lower applied potential is favourable for higher sensor selectivity, the linear sweep voltammetry was used in all subsequent experiments.

## Characterization of 2LanMEF∆3-41-197-BLA-based bioelectrodes

Electrochemical experiments were conducted using an electrochemical workstation (ECO Chemie Autolab PASTAT 10) and GPES 4.9 (General Purpose Electrochemical System) software. All potentials were measured using a BASi Ag/AgCl/KCl (3 M) reference electrode, and a graphite slab was used as a counter electrode. The lab-made electrodes based on carbon paper (Spectracarb 2050L-1050) modified by copper tape were used as the working electrode. Electrochemical measurements of 2LanMEF∆3-41-197-BLA-chitosan/alginate composite bioelectrodes were carried out in a 2 mL miniaturized electrochemical cell using linear sweep voltammetry in the presence of increasing $YCl_3$ concentrations. Conditions: 25 mM Tris-HCl buffer, pH 7.2; 300 µM UW154; scan rate 50 mV/s vs. Ag/AgCl/3 M KCl reference electrode at room temperature avoiding direct light. The calibration data collected for the sensor's output was taken at +0.365 V vs. Ag/AgCl/3 MKCl reference.

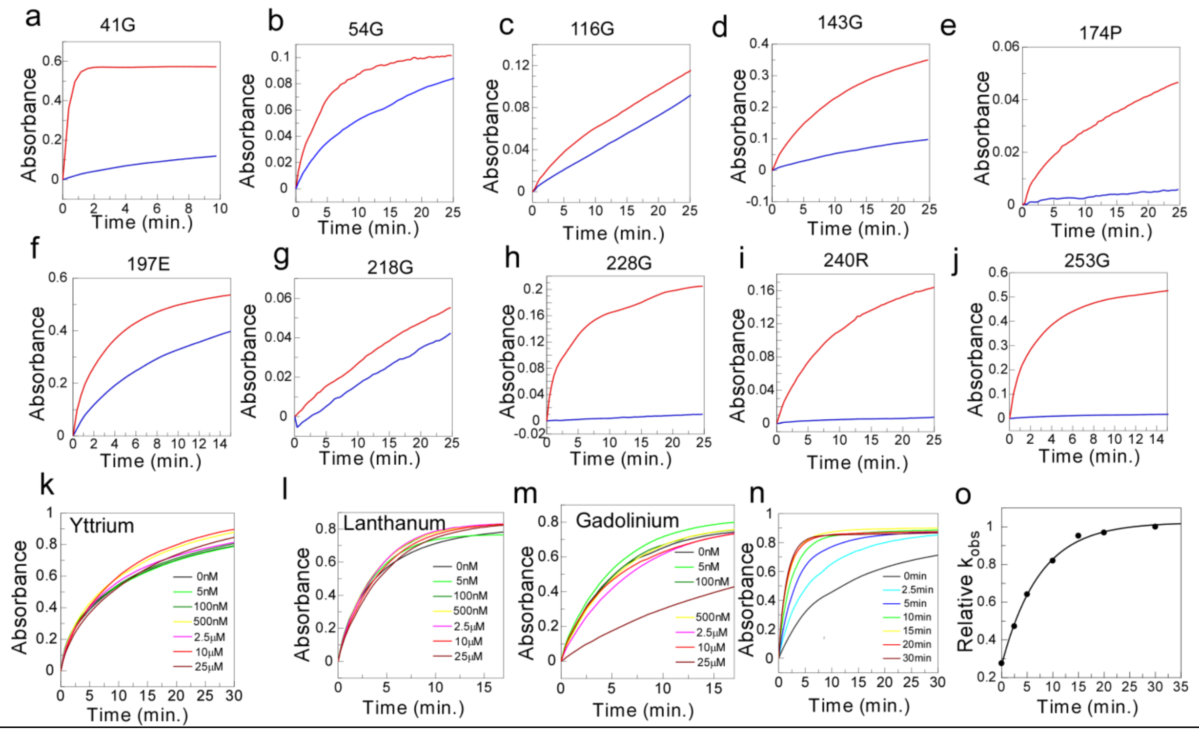


*Supplementary figure 1.* Activity analysis of ten LanM-BLA chimeras. In the experiments 25 nM solutions of purified LanM-BLA chimeras (a-j) was incubated for 15 minutes in the presence (red trace) or absence (blue trace) of 0.5µM of $YCl_3$ after which the reaction mixture was supplemented with 50 µM of UW154 and changes in absorption at 520 nm were followed for the indicated periods of time. The identities of the chimeras are indicated above the plots. (k-m) Activity analysis of a 5 nM solution of wild type TEM-18 BLA in the presence of increasing concentrations of REE chloride salts. (n) Time dependent changes

in activity of 25 nM solution of LanM-41-BLA chimera upon exposure to saturating (250nM) concentrations of $YCl_3$. (o) a plot of the data shown in (n) fitted to a single exponential activation rate.

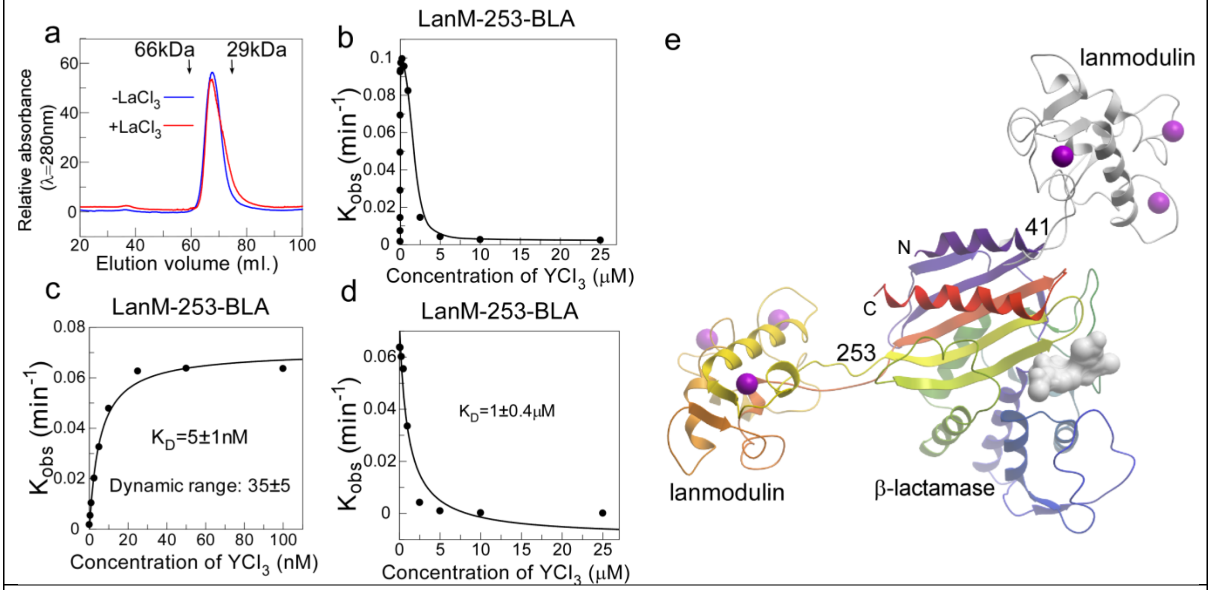


*Supplementary figure 2.* Analysis of LanM-41-BLA and LanM-253-BLA chimeras. (a) Elution profile of 2mg of LanM-41-BLA chimera separated on Superdex 75 16/60 gel filtration column equilibrated with 2 0mM Tris pH7.2, 100mM NaCl and the absence (blue line) or presence of 1μM $LaCl_3$. The position of molecular weight markers used for calibration of the column are indicated by the arrows. The molecular weight of LanM-41-BLA chimera was calculated as 42kDa that closely corresponds to a monomer. (b) A plot of $k_{obs}$ values extracted from the absorbance time traces of 10 nM of LanM-253-BLA and 50μM of UW154 obtained at the indicated concentration of $YCl_3$. (c) A plot of $k_{obs}$ values in the 0-100nM $YCl_3$ concentration range fitted to a quadratic equation resulting in an apparent $K_D$ of 5nM. (d) As in (c) but analysing biosensor's response in 0.1-25 μM range leading to an apparent $K_D$ of 1 μM. (e) Superimposed models of LanM-41-BLA (white) and LanM-253-BLA chimera (coloured spectrally form N- to C- termini) based on PDB structures 6mi5 and 3gmw displayed in ribbon representation. The Lns ions are displayed as magenta balls and the active site of BLA is marked by an inhibitor imported from PDB:6c79. The insertion sites on BLA are marked by the replaced residue number.

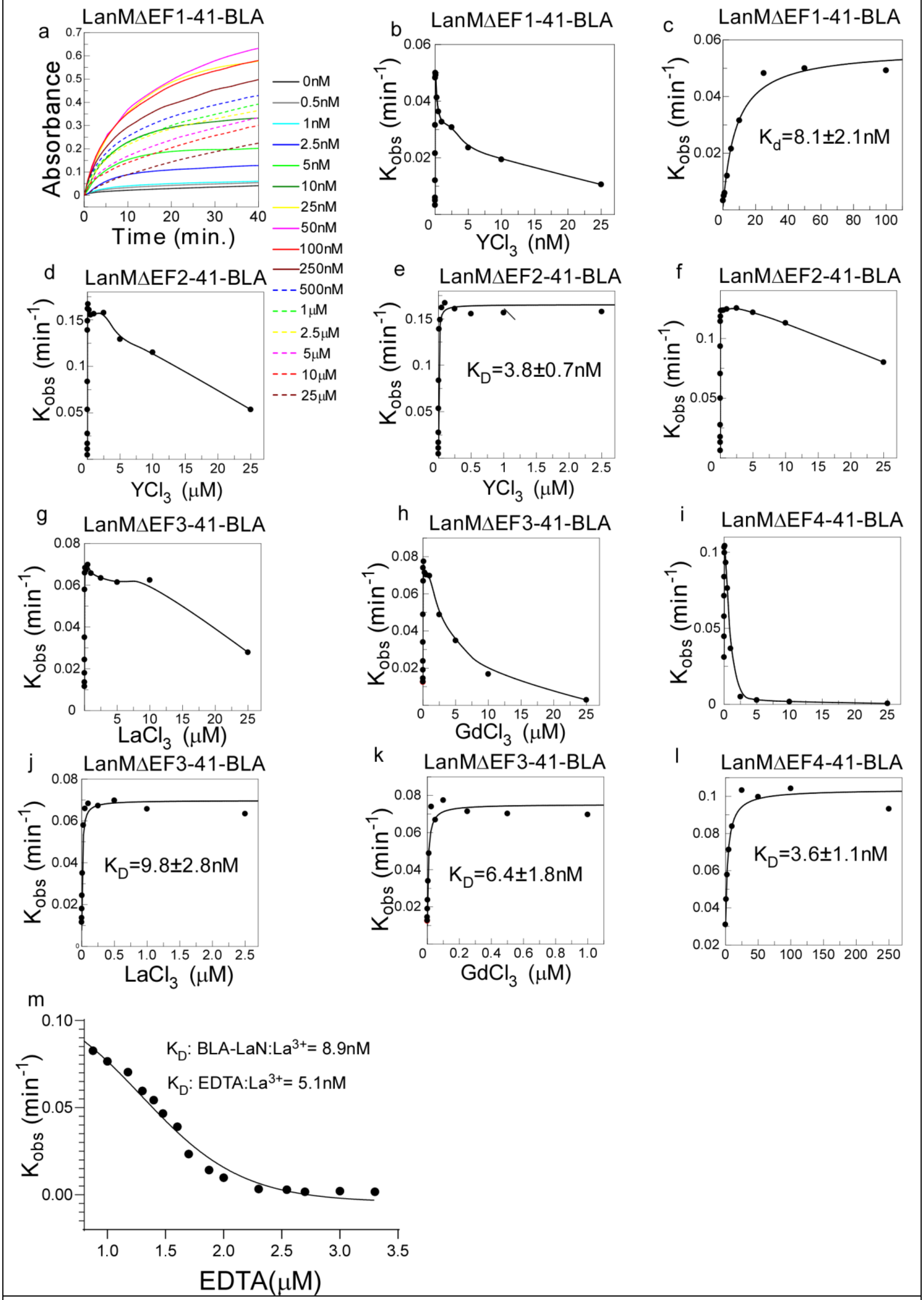


*Supplementary figure 3.* Activity analysis of LanM-41-BLA chimera with mutates EF hands. (a) Change of UW154 absorption in the presence of 10 nM of LanMΔEF1-41-BLA and

increasing concentrations of $YCl_3$. The colour coding of the traces is shown on the left of the plot. (b) A plot of $k_{obs}$ values extracted from the absorbance time traces shown in (a). (c) A plot of $k_{obs}$ values in the 0-100nM $YCl_3$ concentration range from (a) fitted to a quadratic equation resulting in an apparent $K_D$ of 8.1 nM. (d) as in (b) but using LanMΔEF2 -41-BLA chimera. (e) as in (c) but using LanMΔEF2-41-BLA chimera. (f) as in (a) but using LanMΔEF3-41-BLA chimera. (g) A plot of $k_{obs}$ values extracted from the absorbance traces of UW154 in the presence of 10nM of LanMΔEF3-41-BLA and the increasing concentrations of $LaCl_3$ (h) as in (g) but using $GdCl_3$ as a titrant (i) as in (b) but using LanMΔEF4-41-BLA chimera. (j) Fit of the $k_{obs}$ values from (g) to a quadratic equation resulting in a $K_D$ value of 10nM. (k) as in J but for $GdCl_3$ as titrant. (l) as in (c) but using LanMΔEF4-41BLA chimera as a biosensor. (m) A plot of the $k_{obs}$ values obtained by titrating a mixture of 25 nM LanMΔEF3-41-BLA chimera with 25 nM of $LaCl_3$ with the increasing concentrations of EDTA. The data was fitted as described in Methods sections.

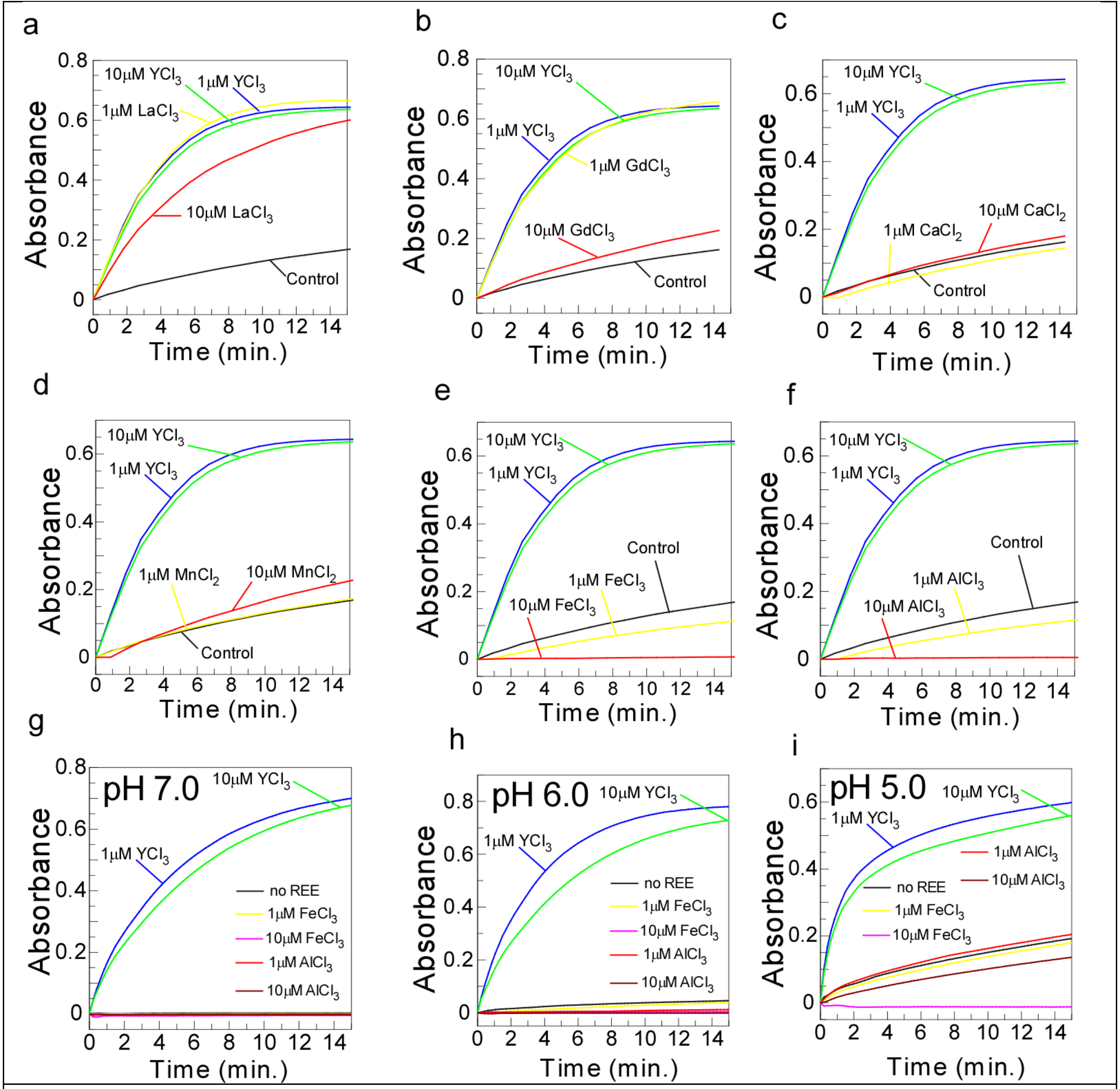


*Supplementary figure 4.* Specificity of LanMΔEF3-41-BLA chimera towards different metal ions. (a) Time-resolved changes in absorbance of UW154 in the presence of 10 nM of LanMΔEF3-41-BLA chimera and indicated concentrations of $LaCl_3$ metal. In these and other experiments $YCl_3$ was used as positive reference while reaction without metal ions (black line) was used as negative control. (b) as in (a) but using $GdCl_3$. (c) as in (a) but using $CaCl_2$ as titrant. (d) Using $MnCl_2$ as titrant. (e) Using $FeCl_3$ as titrant (f) Using $AlCl_3$ as titrant. (g-h) testing the response of the chimera towards different ions at different pH. Conditions used were as in (a) but using 25 nM of LanMΔEF3-41G-BLA chimera.

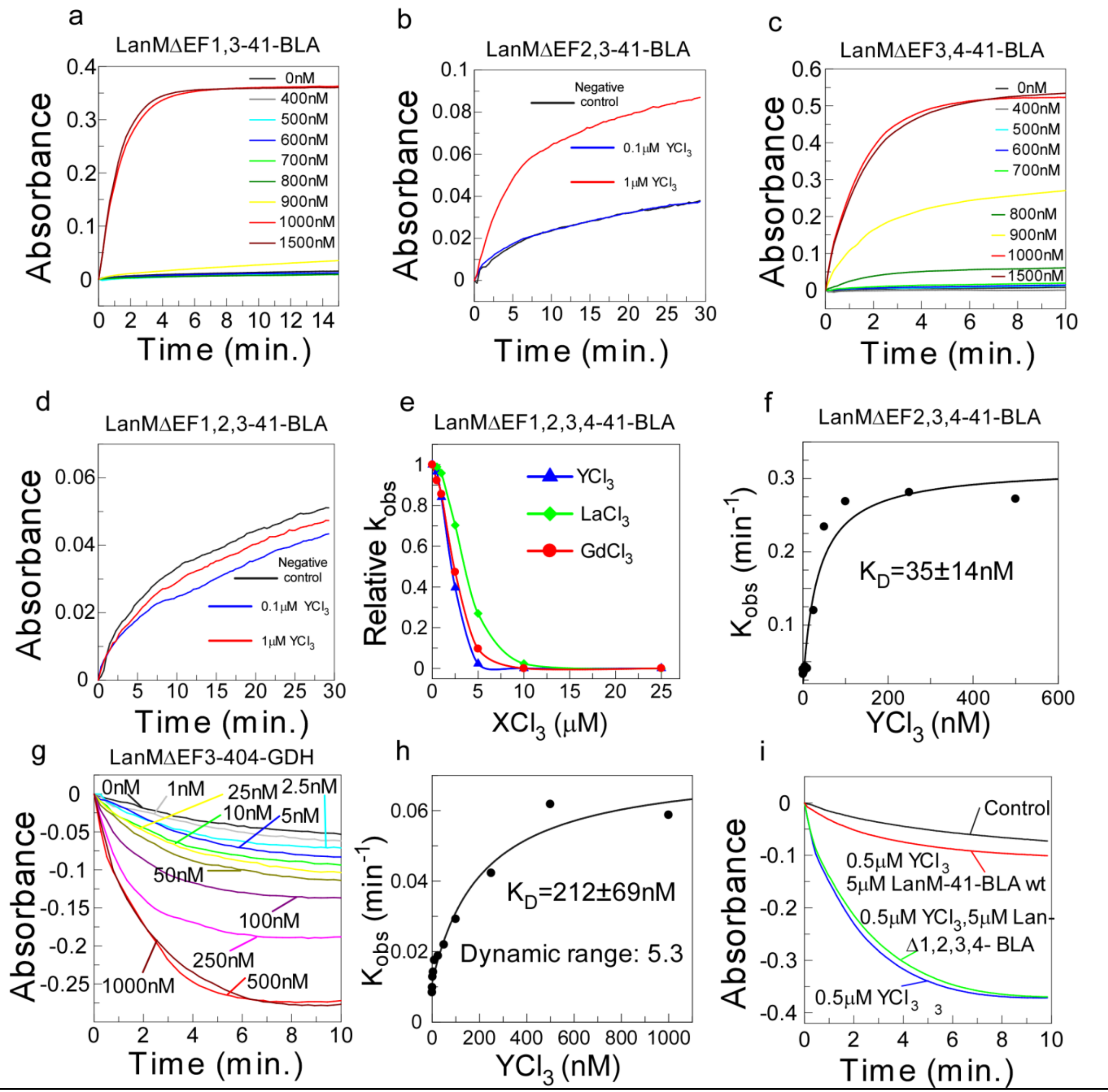


*Supplementary figure 5.* Analysis of different LanM-based biosensors. (a) Changes in β-lactamase activity of 25 nM solution of LanMΔEF1,3-41-BLA in response to the indicated concentrations of $YCl_3$. (b) As in (a) but using LanMΔEF2,3-41-BLA. (c) as in (a) but using LanMΔEF3,4-41-BLA. (d) As in (a) but using LanMΔEF1,2,3-41-BLA. (e) A plot of $k_{obs}$ values of reactions containing 100nM of LanMΔEF 1,2,3,4-41-BLA chimera and 50μM of UW154 and indicated concentrations of lanthanide chlorite salts. (f) A plot of $k_{obs}$ values extracted from the absorbance time traces shown in Figure 2b were 25 nM of the biosensor with a single intact EF-hand 1 (LanMBLAΔ2,3,4-41-BLA) were titrated with the increasing concentrations of $YCl_3$. (g) Absorbance changes of 10nM solution of LanMEFΔ3-404-GDH chimera 20mM Tris pH7.4, 100mM NaCl, 100μM $CalCl_2$, 600μM PMS and 60μM DCPIP in the presence of indicated concentrations of $YCl_3$. Fit of the data from (g) to a quadratic equation. (i) Using LanMEFΔ3-404-GDH to assess Lns binding capacity of LanMEFΔ1,2,3,4-41-BLA. In

the experiment response of 10nM LanMEFΔ3-404-GDH to $YCl_3$ was assessed in the presence or absence of 50 fold excess of LanMEFΔEF1,2,3,4-41-BLA chimera.

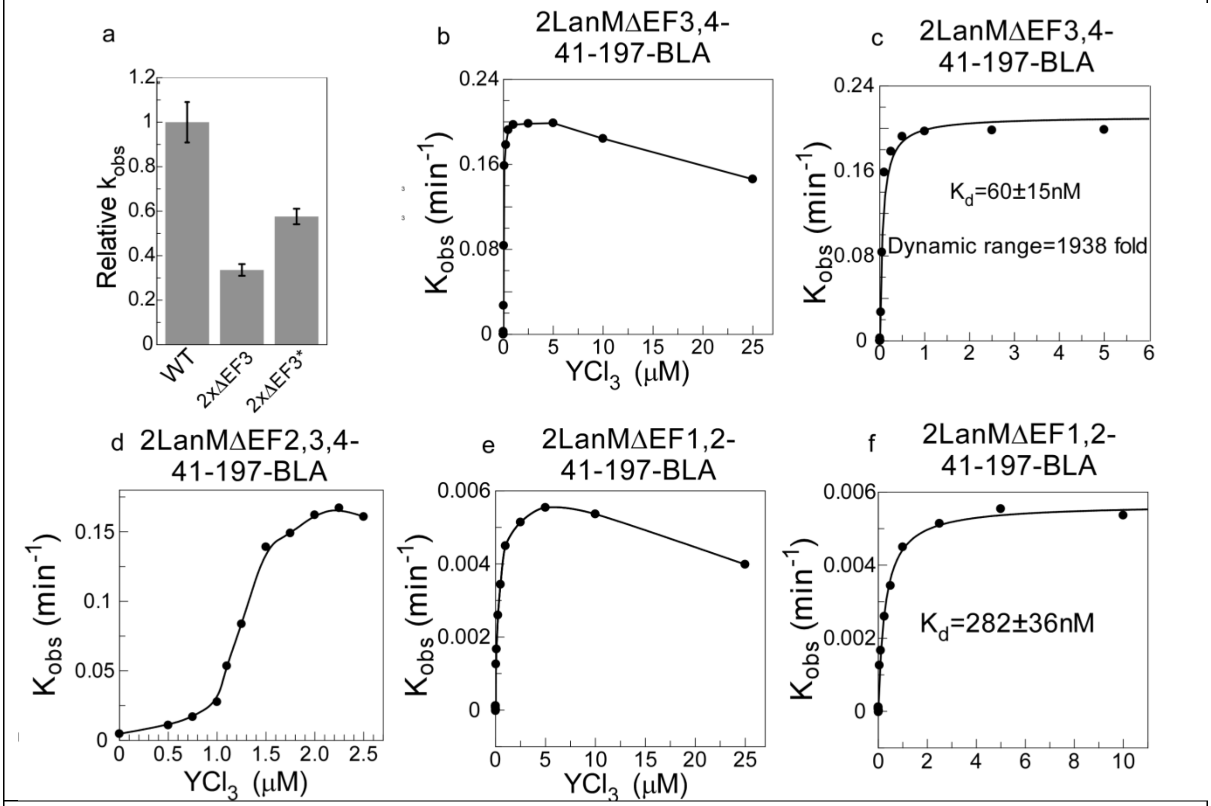


*Supplementary figure 6.* Characterisation of LanM-BLA chimeras with double LanM insertions. (a) Relative activity of wild type β-lactamase, 2LanMΔEF3-41-LanM 197-BLA chimera and its thermostabilised variant. In the analysis the $k_{obs}$ calculated by fitting the initial reaction rates of samples containing 25nM of each enzyme, 50μM UW154 and 1μM $YCl_3$. (b) Plot of $k_{obs}$ values obtaining by titrating 25nM of 2LanMΔEF3,4-41-197-BLA with increasing concentrations of $YCl_3$. (c) Fit of the titration data between 0 and 5μM shown in (b) to a quadratic equation resulting in a $K_D$ of 60nM. (d) Plot of $k_{obs}$ values obtained by titrating 25nM of 2LanMEFΔ2,3,4-41-197-BLA with increasing concentrations of $YCl_3$. (e) as in (d) but using 2LanMEFΔ1,2-41-197-BLA. (f) Fit of the $k_{obs}$ values from (e) to a quadratic equation.

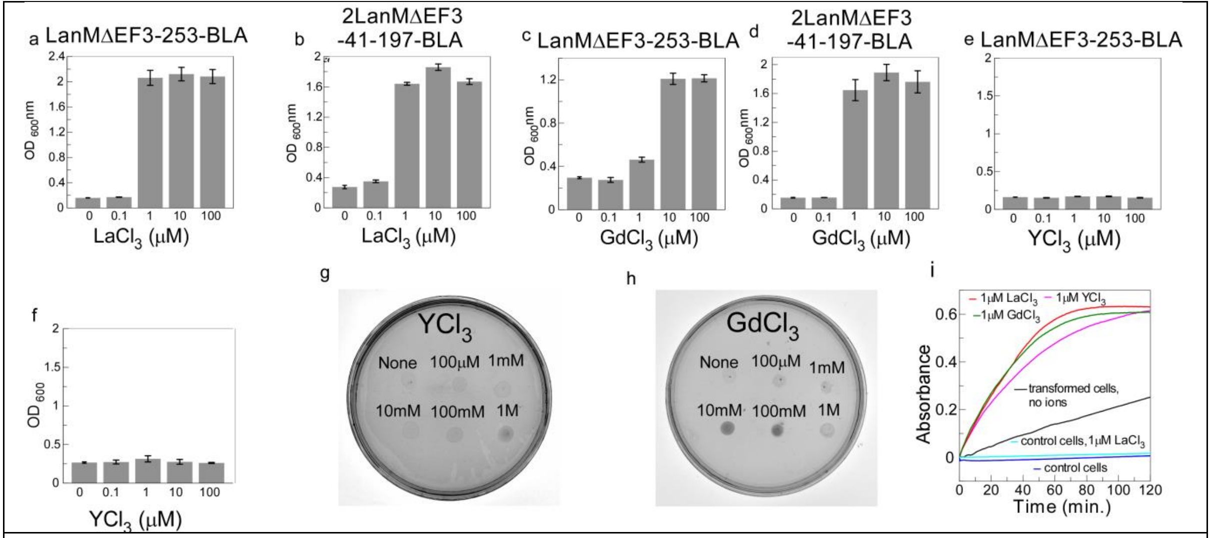


*Supplementary figure 7.* Analysis of ampicillin resistance of *E.coli* strains DH5α constitutively expressing various LanM-BLA chimeras under the control of a constitutive promoter. (a) A plot of suspension culture density of *E. coli* DH5α transformed with LanMΔEF3-253G-BLA grown at the indicated concentrations of $LaCl_3$. (b) As in (a) but using 2LanMΔEF3-41-197-BLA construct. (c) As in (a) but growing cells in the presence of $GdCl_3$. (d) as in (b) but growing cells in the presence of $GdCl_3$. (e) As in (a) but growing cells in the presence of $YCl_3$. (f) as in (b) but growing cells in the presence of $YCl_3$. (g) An agar plate containing ampicillin and chloramphenicol antibiotics seeded with *E.coli* strain DH5α expressing 2LanMΔEF3-41-197-BLA chimera. After bacterial plating 2 μL of indicated concentration of $YCl_3$ were spotted at the indicated positions and bacterial growth was allowed to develop overnight. (h) As in (g) but using indicated concentrations of $GdCl_3$. (i) Spectroscopic detection of REE-dependent β-lactamase activity of *E. coli* cells expressing 2LanMΔEF3-41-197-BLA. In these experiments BL21(DE3) cells transformed with pET28a-2LanMΔEF3-41-197-BLA were induced with 0.3mM IPTG. After an expression period (overnight), 2mL culture was spun down and washed 8 times with fresh LB medium and then suspended with 2mL buffer containing 20mM Tris pH7.2, 100mM NaCl. 100μL aliquots were withdrawn and diluted to 1mL with the same buffer supplemented with 50μM UW154 and indicated concentrations of REE. The change in absorbance at 520nm was recorded for the indicated period.

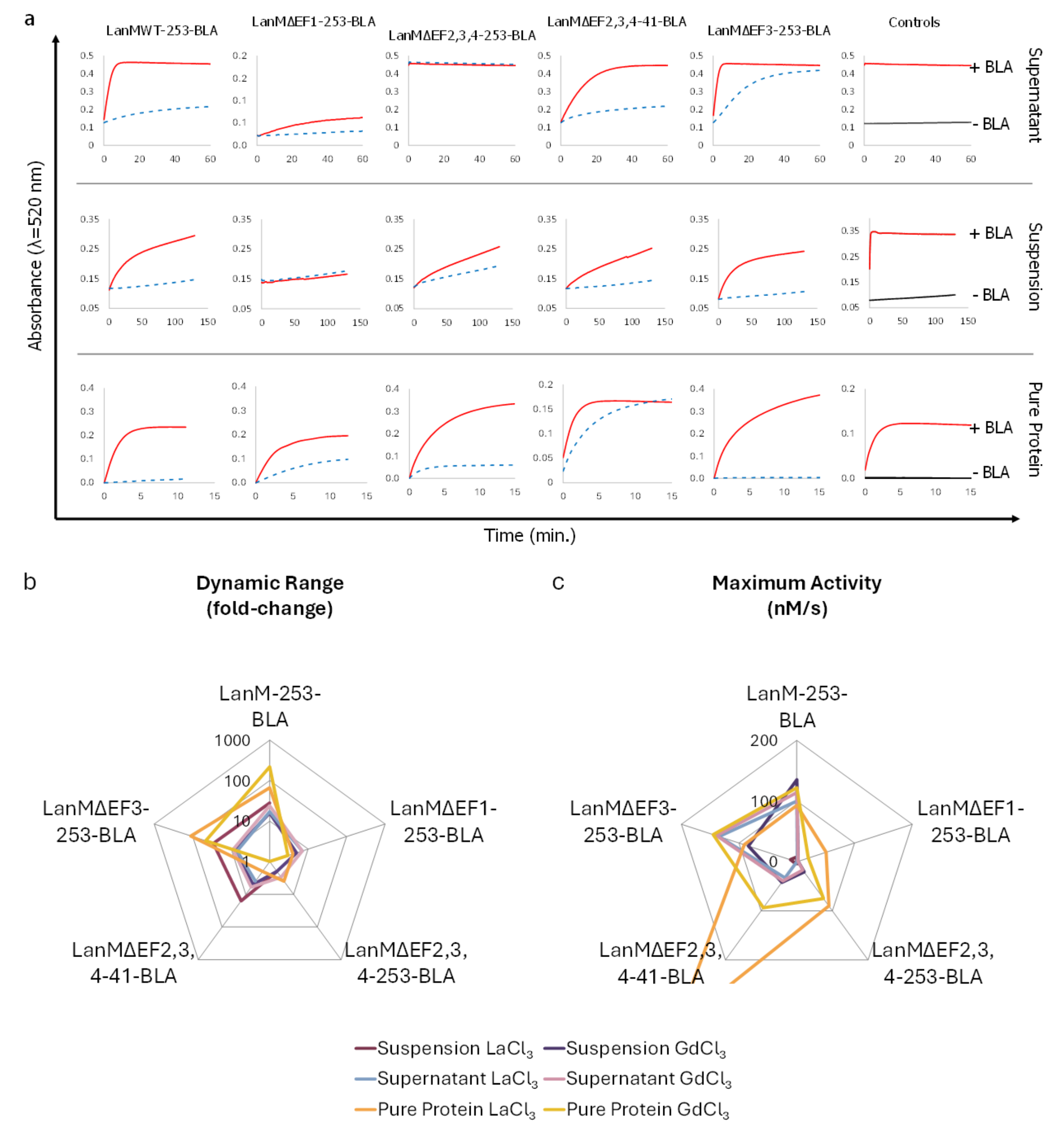


*Supplementary figure 8.* Monitoring LanM biosensor activity in suspension of living *E.coli* cells or cultural supernatants (a) Comparison of Lns-dependent β-lactamase activity of cultural supernatant of NEBExpress *E.coli* culture transformed with modified pCola-Duet vector encoding indicated LanM-based biosensors (top row) or culture suspension (middle row) or the corresponding biosensors introduced into the reaction as recombinant proteins. The blue dashed trace represents the background activity of the system while the red trace represents activity in the presence of 500nM $LaCl_3$. Prior to experiments protein expression was induced overnight by addition of IPTG. The negative control represents cells expressing Cog3 protein while cells expressing wild type TEM-18 β-lactamase served as positive controls in cellular and suspension experiments. (b) A radar plot of dynamic ranges of

different biosensors measured using purified protein, cultural supernatant or cell suspension. (c) As in (b) but plotting the catalytic activity of biosensors in the presence of 500nM $LaCl_3$.

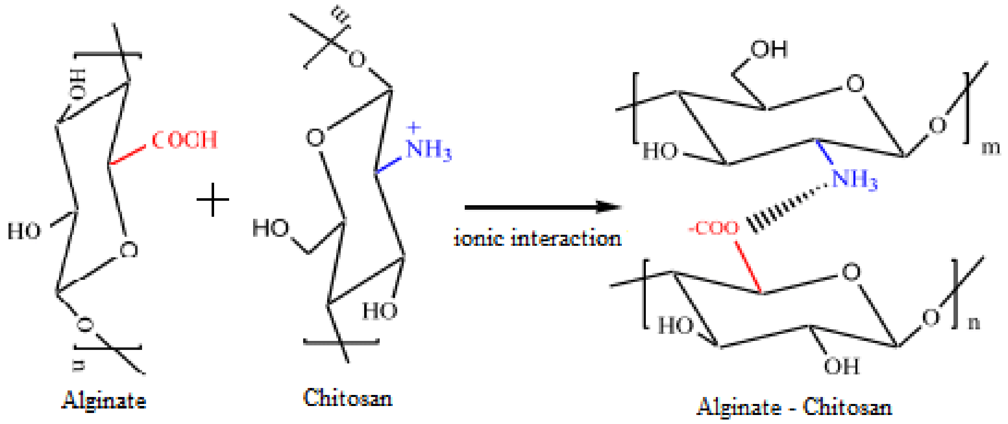


*Supplementary figure 9.* Schematic of ionic interaction between chitosan and alginate resulting in the formation of a strong chitosan/alginate composite film.

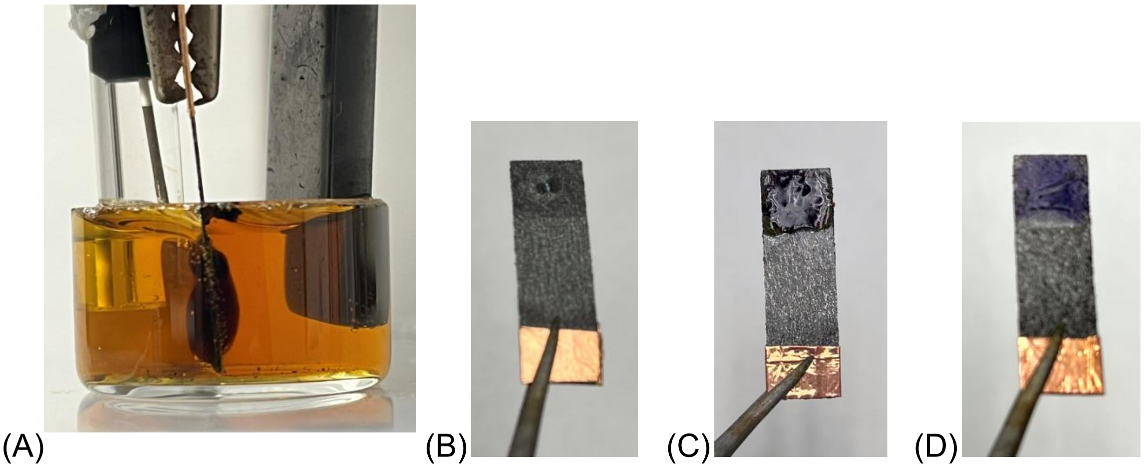


*Supplementary figure 10.* Development and testing of LanM-BLA based bioelectrodes. (a) Photograph of an electrochemical cell filled with reagent solution (25 mM Tris-HCl buffer, pH 7.2; 300 μM UW154; 100 μM $YCl_3$) with integrated BLA-2LaM-chitosan/alginate composite bioelectrode. (b) The BLA-2LaM-chitosan/alginate composite bioelectrode before assay. (c) The same electrode after enzymatic UW154 hydrolysis. (d) The same electrode at in a dried state.

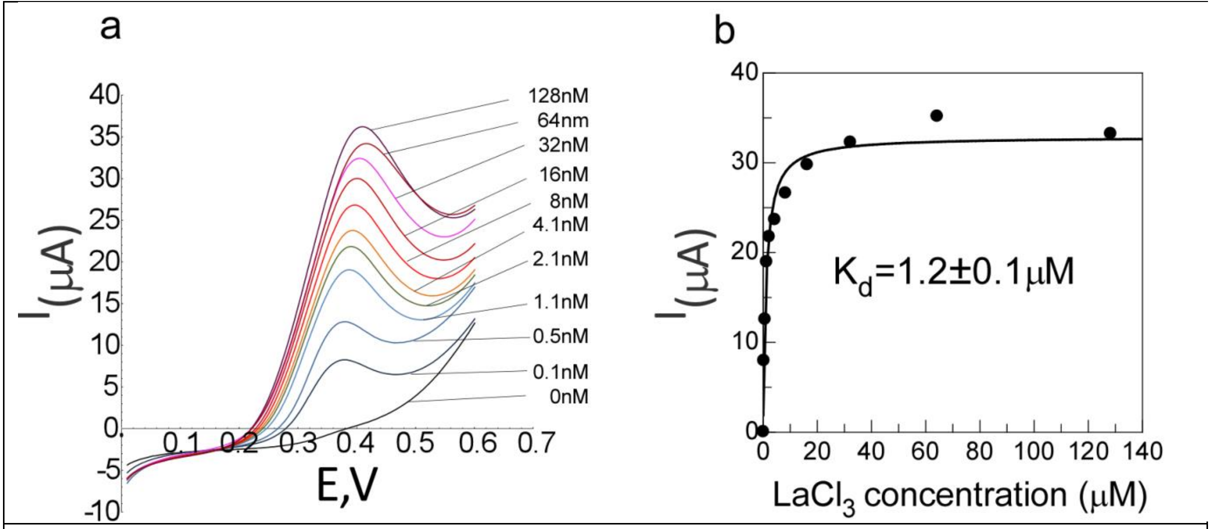


*Supplementary figure 11.* Electrochemical analysis of LanM-BLA based bioelectrodes (a) A typical linear sweep voltammograms of 2LanMΔ3-41-LanM 197-BLA-chitosan/alginate composite bioelectrode in the presence of 300 μM UW154 and increasing $LaCl_3$ concentrations. The titration data collected for the sensor's output taken at +0.365 V vs. Ag/AgCl/3 M KCl reference. (b) Fit of the titration data shown in (a) to a quadratic equation leading to an apparent $K_d$ value of 1.2 μM.

**Supplementary Table 1**. Performance parameters of LanMLanM-reporter chimeras

| Construct name | Lanmodulin insertion site | $k_{obs}$ $min^{-1}$ at 25 nM of enzyme with 0.5μM $YCl_3$ | Dynamic range |
|---|---|---|---|
| wt BLA | | 1.87±0.17 | |
| 1 | 41G | 0.25±0.04 | 62.2±7.8 |
| 2 | 54G | 0.007±0.001 | |
| 3 | 116G | 0.008±0.001 | 1.8±0.3 |
| 4 | 143G | 0.029±0.004 | 4.9±0.6 |
| 5 | 174P | 0.004±0.001 | 7.7±0.9 |
| 6 | 197E | 0.15±0.02 | 2.3±0.4 |
| 7 | 218G | 0.003±0.0008 | |
| 8 | 228G | 0.05±0.007 | 89±12 |
| 9 | 240R | 0.02±0.003 | 23±3 |
| 10 | 253G | 0.16±0.04 | 35.4±4.8 |
| 11 | 41G ΔEF1 | 0.13±0.03 | 15±3 |
| 12 | 41G ΔEF2 | 0.41±0.04 | 35±5 |
| 13 | 41G ΔEF3 | 0.32±0.03 | 20±3 |
| 14 | 41G ΔEF4 | 0.26±0.03 | 3.4±0.3 |
| 15 | 41G ΔEF1,3 | 0.23±0.03 | 42±4 |
| 16 | 41G ΔEF2,3 | 0.004±0.001 | 2.8±0.4 |
| 17 | 41G ΔEF3,4 | 0.28±0.04 | 128±15 |
| 18 | 41G ΔEF1,2,3 | 0.004±0.001 | |
| 19 | 41G ΔEF2,3,4 | 0.17±0.02 | 7.2±1.1 |
| 20 | 41G ΔEF1,2,3,4 | 0.14±0.02 | |
| 21 | BLA-2LanM 41G 197E ΔEF3 | 0.63±0.05 | 2941±341 |
| 22 | thermostable BLA-2LanM 41G 197E ΔEF3 | 1.08±0.07 | 1503±273 |
| 24 | BLA-2LanM 41G 197E ΔEF1,3 | 0.006±0.001 | 79±9 |
| 25 | BLA-2LanM 41G 197E ΔEF3,4 | 0.20±0.02 | 1938±328 |
| 26 | BLA-2LanM 41G 197E ΔEF2,3,4 | 0.28±0.02 | 7.1±0.6 |
| 27 | LanMΔ3-404-GDH | 0.06 (10nM enzyme) | 7.2±1.1 |

**Supplementary Table 2**. Sequences of constructs used in this study. Where relevant functional domains or linker elements are highlighted using color, bolding or underlining.

| Construct | Sequence |
|---|---|
| Lanmodulin-β-lactamase-41G | DHPETLVKVKDAEDQLGSGVDIAAFDPDKDGTIDLKEALAAGSAAFDKLDPDKDGTLDAKELKGRVSEADLKKLDPDNDGTLDKKEYLAAVEAQFKAANPDNDGTIDARELASPAGSALVNLIRGSGARVGYIELDLNSGKILESFRPEERFPMMSTFKVLLCGAVLSRVDAGQEQLGRRIHYSQNDLVEYSPVTEKHLTDGMTVRELCSAAITMSDNTAANLLLTTIGGPKELTAFLHNMGDHVTRLDRWEPELNEAIPNDERDTTMPVAMATTLRKLLTGELLTLASRQQLIDWMEADKVAGPLLRSALPAGWFIADKSGAGERGSRGIIAALGPDGKPSRIVVIYTTGSQATMDERNRQIAEIGASLIKHWGKLAAALEHHHHHH |
| Lanmodulin-β-lactamase-54G | DHPETLVKVKDAEDQLGARVGYIELDLNSGSGVDIAAFDPDKDGTIDLKEALAAGSAAFDKLDPDKDGTLDAKELKGRVSEADLKKLDPDNDGTLDKKEYLAAVEAQFKAANPDNDGTIDARELASPAGSALVNLIRGSGKILESFRPEERFPMMSTFKVLLCGAVLSRVDAGQEQLGRRIHYSQNDLVEYSPVTEKHLTDGMTVRELCSAAITMSDNTAANLLLTTIGGPKELTAFLHNMGDHVTRLDRWEPELNEAIPNDERDTTMPVAMATTLRKLLTGELLTLASRQQLIDWMEADKVAGPLLRSALPAGWFIADKSGAGERGSRGIIAALGPDGKPSRIVVIYTTGSQATMDERNRQIAEIGASLIKHWGKLAAALEHHHHHH |
| Lanmodulin-β-lactamase-116G | DHPETLVKVKDAEDQLGARVGYIELDLNSGKILESFRPEERFPMMSTFKVLLCGAVLSRVDAGQEQLGRRIHYSQNDLVEYSPVTEKHLTDGSGVDIAAFDPDKDGTIDLKEALAAGSAAFDKLDPDKDGTLDAKELKGRVSEADLKKLDPDNDGTLDKKEYLAAVEAQFKAANPDNDGTIDARELASPAGSALVNLIRGSGMTVRELCSAAITMSDNTAANLLLTTIGGPKELTAFLHNMGDHVTRLDRWEPELNEAIPNDERDTTMPVAMATTLRKLLTGELLTLASRQQLIDWMEADKVAGPLLRSALPAGWFIADKSGAGERGSRGIIAALGPDGKPSRIVVIYTTGSQATMDERNRQIAEIGASLIKHWGKLAAALEHHHHHH |
| Lanmodulin-β-lactamase-143G | DHPETLVKVKDAEDQLGARVGYIELDLNSGKILESFRPEERFPMMSTFKVLLCGAVLSRVDAGQEQLGRRIHYSQNDLVEYSPVTEKHLTDGMTVRELCSAAITMSDNTAANLLLTTIGSGVDIAAFDPDKDGTIDLKEALAAGSAAFDKLDPDKDGTLDAKELKGRVSEADLKKLDPDNDGTLDKKEYLAAVEAQFKAANPDNDGTIDARELASPAGSALVNLIRGSGGPKELTAFLHNMGDHVTRLDRWEPELNEAIPNDERDTTMPVAMATTLRKLLTGELLTLASRQQLIDWMEADKVAGPLLRSALPAGWFIADKSGAGERGSRGIIAALGPDGKPSRIVVIYTTGSQATMDERNRQIAEIGASLIKHWGKLAAALEHHHHHH |
| Lanmodulin-β-lactamase-174P | DHPETLVKVKDAEDQLGARVGYIELDLNSGKILESFRPEERFPMMSTFKVLLCGAVLSRVDAGQEQLGRRIHYSQNDLVEYSPVTEKHLTDGMTVRELCSAAITMSDNTAANLLLTTIGGPKELTAFLHNMGDHVTRLDRWEPELNEAIGSGVDIAAFDPDKDGTIDLKEALAAGSAAFDKLDPDKDGTLDAKELKGRVSEADLKKLDPDNDGTLDKKEYLAAVEAQFKAANPDNDGTIDARELASPAGSALVNLIRGSGNDERDTTMPVAMATTLRKLLTGELLTLASRQQLIDWMEADKVAGPLLRSALPAGWFIADKSGAGERGSRGIIAALGPDGKPSRIVVIYTTGSQATMDERNRQIAEIGASLIKHWGKLAAALEHHHHHH |
| Lanmodulin-β-lactamase-197E | DHPETLVKVKDAEDQLGARVGYIELDLNSGKILESFRPEERFPMMSTFKVLLCGAVLSRVDAGQEQLGRRIHYSQNDLVEYSPVTEKHLTDGMTVRELCSAAITMSDNTAANLLLTTIGGPKELTAFLHNMGDHVTRLDRWEPELNEAIPNDERDTTMPVAMATTLRKLLTGGSGVDIAAFDPDKDGTIDLKEALAAGSAAFDKLDPDKDGTLDAKELKGRVSEADLKKLDPDNDGTLDKKEYLAAVEAQFKAANPDNDGTIDARELASPAGSALVNLIRGSGLLTLASRQQLIDWMEADKVAGPLLRSALPAGWFIADKSGAGE |

| | |
|---|---|
| | RGSRGIIAALGPDGKPSRIVVIYTTGSQATMDERNRQIAEIGASLIKHWGKLAAAL EHHHHHH |
| Lanmodulin-β-lactamase-218G | DHPETLVKVKDAEDQLGARVGYIELDLNSGKILESFRPEERFPMMSTFKVLLCGA VLSRVDAGQEQLGRRIHYSQNDLVEYSPVTEKHLTDGMTVRELCSAAITMSDNT AANLLLTTIGGPKELTAFLHNMGDHVTRLDRWEPELNEAIPNDERDTTMPVAM ATTLRKLLTGELLTLASRQQLIDWMEADKVAGSGVDIAAFDPDKDGTIDLKEALA AGSAAFDKLDPDKDGTLDAKELKGRVSEADLKKLDPDNDGTLDKKEYLAAVEAQ FKAANPDNDGTIDARELASPAGSALVNLIRGSGPLLRSALPAGWFIADKSGAGE RGSRGIIAALGPDGKPSRIVVIYTTGSQATMDERNRQIAEIGASLIKHWGKLAAAL EHHHHHH |
| Lanmodulin-β-lactamase-228G | DHPETLVKVKDAEDQLGARVGYIELDLNSGKILESFRPEERFPMMSTFKVLLCGA VLSRVDAGQEQLGRRIHYSQNDLVEYSPVTEKHLTDGMTVRELCSAAITMSDNT AANLLLTTIGGPKELTAFLHNMGDHVTRLDRWEPELNEAIPNDERDTTMPVAM ATTLRKLLTGELLTLASRQQLIDWMEADKVAGPLLRSALPAGSGVDIAAFDPDKD GTIDLKEALAAGSAAFDKLDPDKDGTLDAKELKGRVSEADLKKLDPDNDGTLDK KEYLAAVEAQFKAANPDNDGTIDARELASPAGSALVNLIRGSGWFIADKSGAGE RGSRGIIAALGPDGKPSRIVVIYTTGSQATMDERNRQIAEIGASLIKHWGKLAAAL EHHHHHH |
| Lanmodulin-β-lactamase-240G | DHPETLVKVKDAEDQLGARVGYIELDLNSGKILESFRPEERFPMMSTFKVLLCGA VLSRVDAGQEQLGRRIHYSQNDLVEYSPVTEKHLTDGMTVRELCSAAITMSDNT AANLLLTTIGGPKELTAFLHNMGDHVTRLDRWEPELNEAIPNDERDTTMPVAM ATTLRKLLTGELLTLASRQQLIDWMEADKVAGPLLRSALPAGWFIADKSGAGEG SGVDIAAFDPDKDGTIDLKEALAAGSAAFDKLDPDKDGTLDAKELKGRVSEADLK KLDPDNDGTLDKKEYLAAVEAQFKAANPDNDGTIDARELASPAGSALVNLIRGS GSRGIIAALGPDGKPSRIVVIYTTGSQATMDERNRQIAEIGASLIKHWGKLAAALE HHHHHH |
| Lanmodulin-β-lactamase-253G | DHPETLVKVKDAEDQLGARVGYIELDLNSGKILESFRPEERFPMMSTFKVLLCGA VLSRVDAGQEQLGRRIHYSQNDLVEYSPVTEKHLTDGMTVRELCSAAITMSDNT AANLLLTTIGGPKELTAFLHNMGDHVTRLDRWEPELNEAIPNDERDTTMPVAM ATTLRKLLTGELLTLASRQQLIDWMEADKVAGPLLRSALPAGWFIADKSGAGER GSRGIIAALGPDGSGVDIAAFDPDKDGTIDLKEALAAGSAAFDKLDPDKDGTLDA KELKGRVSEADLKKLDPDNDGTLDKKEYLAAVEAQFKAANPDNDGTIDARELAS PAGSALVNLIRGSGKPSRIVVIYTTGSQATMDERNRQIAEIGASLIKHWGKLAAA LEHHHHHH |
| Lanmodulin(EF1 mutant)-β-lactamase-41G | DHPETLVKVKDAEDQLGSGVDIAAFDPSKSGTIDLKEALAAGSAAFDKLDPDKD GTLDAKELKGRVSEADLKKLDPDNDGTLDKKEYLAAVEAQFKAANPDNDGTIDA RELASPAGSALVNLIRGSGARVGYIELDLNSGKILESFRPEERFPMMSTFKVLLCG AVLSRVDAGQEQLGRRIHYSQNDLVEYSPVTEKHLTDGMTVRELCSAAITMSD NTAANLLLTTIGGPKELTAFLHNMGDHVTRLDRWEPELNEAIPNDERDTTMPV AMATTLRKLLTGELLTLASRQQLIDWMEADKVAGPLLRSALPAGWFIADKSGA GERGSRGIIAALGPDGKPSRIVVIYTTGSQATMDERNRQIAEIGASLIKHWGKLA AALEHHHHHH |
| Lanmodulin(EF2 mutant)-β-lactamase-41G | DHPETLVKVKDAEDQLGSGVDIAAFDPDKDGTIDLKEALAAGSAAFDKLDPSKS GTLDAKELKGRVSEADLKKLDPDNDGTLDKKEYLAAVEAQFKAANPDNDGTIDA RELASPAGSALVNLIRGSGARVGYIELDLNSGKILESFRPEERFPMMSTFKVLLCG AVLSRVDAGQEQLGRRIHYSQNDLVEYSPVTEKHLTDGMTVRELCSAAITMSD NTAANLLLTTIGGPKELTAFLHNMGDHVTRLDRWEPELNEAIPNDERDTTMPV AMATTLRKLLTGELLTLASRQQLIDWMEADKVAGPLLRSALPAGWFIADKSGA GERGSRGIIAALGPDGKPSRIVVIYTTGSQATMDERNRQIAEIGASLIKHWGKLA AALEHHHHHH |

| | |
|---|---|
| Lanmodulin(EF3 mutant)-β-lactamase-41G | DHPETLVKVKDAEDQLGSGVDIAAFDPDKDGTIDLKEALAAGSAAFDKLDPDKDGTLDAKELKGRVSEADLKKLDPSNSGTLDKKEYLAAVEAQFKAANPDNDGTIDARELASPAGSALVNLIRGSGARVGYIELDLNSGKILESFRPEERFPMMSTFKVLLCGAVLSRVDAGQEQLGRRIHYSQNDLVEYSPVTEKHLTDGMTVRELCSAAITMSDNTAANLLLTTIGGPKELTAFLHNMGDHVTRLDRWEPELNEAIPNDERDTTMPVAMATTLRKLLTGELLTLASRQQLIDWMEADKVAGPLLRSALPAGWFIADKSGAGERGSRGIIAALGPDGKPSRIVVIYTTGSQATMDERNRQIAEIGASLIKHWGKLAAALEHHHHHH |
| Lanmodulin(EF4 mutant)-β-lactamase-41G | DHPETLVKVKDAEDQLGSGVDIAAFDPDKDGTIDLKEALAAGSAAFDKLDPDKDGTLDAKELKGRVSEADLKKLDPDNDGTLDKKEYLAAVEAQFKAANPSNSGTIDARELASPAGSALVNLIRGSGARVGYIELDLNSGKILESFRPEERFPMMSTFKVLLCGAVLSRVDAGQEQLGRRIHYSQNDLVEYSPVTEKHLTDGMTVRELCSAAITMSDNTAANLLLTTIGGPKELTAFLHNMGDHVTRLDRWEPELNEAIPNDERDTTMPVAMATTLRKLLTGELLTLASRQQLIDWMEADKVAGPLLRSALPAGWFIADKSGAGERGSRGIIAALGPDGKPSRIVVIYTTGSQATMDERNRQIAEIGASLIKHWGKLAAALEHHHHHH |
| Lanmodulin(EF1,3 mutant)-β-lactamase-41G | DHPETLVKVKDAEDQLGSGVDIAAFDPSKSGTIDLKEALAAGSAAFDKLDPDKDGTLDAKELKGRVSEADLKKLDPSNSGTLDKKEYLAAVEAQFKAANPDNDGTIDARELASPAGSALVNLIRGSGARVGYIELDLNSGKILESFRPEERFPMMSTFKVLLCGAVLSRVDAGQEQLGRRIHYSQNDLVEYSPVTEKHLTDGMTVRELCSAAITMSDNTAANLLLTTIGGPKELTAFLHNMGDHVTRLDRWEPELNEAIPNDERDTTMPVAMATTLRKLLTGELLTLASRQQLIDWMEADKVAGPLLRSALPAGWFIADKSGAGERGSRGIIAALGPDGKPSRIVVIYTTGSQATMDERNRQIAEIGASLIKHWGKLAAALEHHHHHH |
| Lanmodulin(EF2,3 mutant)-β-lactamase-41G | DHPETLVKVKDAEDQLGSGVDIAAFDPDKDGTIDLKEALAAGSAAFDKLDPSKSGTLDAKELKGRVSEADLKKLDPSNSGTLDKKEYLAAVEAQFKAANPDNDGTIDARELASPAGSALVNLIRGSGARVGYIELDLNSGKILESFRPEERFPMMSTFKVLLCGAVLSRVDAGQEQLGRRIHYSQNDLVEYSPVTEKHLTDGMTVRELCSAAITMSDNTAANLLLTTIGGPKELTAFLHNMGDHVTRLDRWEPELNEAIPNDERDTTMPVAMATTLRKLLTGELLTLASRQQLIDWMEADKVAGPLLRSALPAGWFIADKSGAGERGSRGIIAALGPDGKPSRIVVIYTTGSQATMDERNRQIAEIGASLIKHWGKLAAALEHHHHHH |
| Lanmodulin(EF3,4 mutant)-β-lactamase-41G | DHPETLVKVKDAEDQLGSGVDIAAFDPDKDGTIDLKEALAAGSAAFDKLDPDKDGTLDAKELKGRVSEADLKKLDPSNSGTLDKKEYLAAVEAQFKAANPSNSGTIDARELASPAGSALVNLIRGSGARVGYIELDLNSGKILESFRPEERFPMMSTFKVLLCGAVLSRVDAGQEQLGRRIHYSQNDLVEYSPVTEKHLTDGMTVRELCSAAITMSDNTAANLLLTTIGGPKELTAFLHNMGDHVTRLDRWEPELNEAIPNDERDTTMPVAMATTLRKLLTGELLTLASRQQLIDWMEADKVAGPLLRSALPAGWFIADKSGAGERGSRGIIAALGPDGKPSRIVVIYTTGSQATMDERNRQIAEIGASLIKHWGKLAAALEHHHHHH |
| Lanmodulin(EF1,2,3 mutant)-β-lactamase-41G | DHPETLVKVKDAEDQLGSGVDIAAFDPSKSGTIDLKEALAAGSAAFDKLDPSKSGTLDAKELKGRVSEADLKKLDPSNSGTLDKKEYLAAVEAQFKAANPDNDGTIDARELASPAGSALVNLIRGSGARVGYIELDLNSGKILESFRPEERFPMMSTFKVLLCGAVLSRVDAGQEQLGRRIHYSQNDLVEYSPVTEKHLTDGMTVRELCSAAITMSDNTAANLLLTTIGGPKELTAFLHNMGDHVTRLDRWEPELNEAIPNDERDTTMPVAMATTLRKLLTGELLTLASRQQLIDWMEADKVAGPLLRSALPAGWFIADKSGAGERGSRGIIAALGPDGKPSRIVVIYTTGSQATMDERNRQIAEIGASLIKHWGKLAAALEHHHHHH |
| Lanmodulin(EF2,3,4 mutant)-β-lactamase-41G | DHPETLVKVKDAEDQLGSGVDIAAFDPDKDGTIDLKEALAAGSAAFDKLDPSKSGTLDAKELKGRVSEADLKKLDPSNSGTLDKKEYLAAVEAQFKAANPSNSGTIDARELASPAGSALVNLIRGSGARVGYIELDLNSGKILESFRPEERFPMMSTFKVLLCGA |

| | |
|---|---|
| | VLSRVDAGQEQLGRRIHYSQNDLVEYSPVTEKHLTDGMTVRELCSAAITMSDNTAANLLLTTIGGPKELTAFLHNMGDHVTRLDRWEPELNEAIPNDERDTTMPVAMATTLRKLLTGELLTLASRQQLIDWMEADKVAGPLLRSALPAGWFIADKSGAGERGSRGIIAALGPDGKPSRIVVIYTTGSQATMDERNRQIAEIGASLIKHWGKLAAALEHHHHHH |
| Lanmodulin(EF1,2,3, 4 mutant)-β-lactamase-41G | DHPETLVKVKDAEDQLGSGVDIAAFDPSKSGTIDLKEALAAGSAAFDKLDPSKSGTLDAKELKGRVSEADLKKLDPSNSGTLDKKEYLAAVEAQFKAANPSNSGTIDARELASPAGSALVNLIRGSGARVGYIELDLNSGKILESFRPEERFPMMSTFKVLLCGAVLSRVDAGQEQLGRRIHYSQNDLVEYSPVTEKHLTDGMTVRELCSAAITMSDNTAANLLLTTIGGPKELTAFLHNMGDHVTRLDRWEPELNEAIPNDERDTTMPVAMATTLRKLLTGELLTLASRQQLIDWMEADKVAGPLLRSALPAGWFIADKSGAGERGSRGIIAALGPDGKPSRIVVIYTTGSQATMDERNRQIAEIGASLIKHWGKLAAALEHHHHHH |
| 2Lanmodulin-β-lactamase-41G 197E | DHPETLVKVKDAEDQLGSGVDIAAFDPDKDGTIDLKEALAAGSAAFDKLDPDKDGTLDAKELKGRVSEADLKKLDPDNDGTLDKKEYLAAVEAQFKAANPDNDGTIDARELASPAGSALVNLIRGSGARVGYIELDLNSGKILESFRPEERFPMMSTFKVLLCGAVLSRVDAGQEQLGRRIHYSQNDLVEYSPVTEKHLTDGMTVRELCSAAITMSDNTAANLLLTTIGGPKELTAFLHNMGDHVTRLDRWEPELNEAIPNDERDTTMPVAMATTLRKLLTGGSGVDIAAFDPDKDGTIDLKEALAAGSAAFDKLDPDKDGTLDAKELKGRVSEADLKKLDPDNDGTLDKKEYLAAVEAQFKAANPDNDGTIDARELASPAGSALVNLIRGSGLLTLASRQQLIDWMEADKVAGPLLRSALPAGWFIADKSGAGERGSRGIIAALGPDGKPSRIVVIYTTGSQATMDERNRQIAEIGASLIKHWGKLAAALEHHHHHH |
| 2Lanmodulin(EF3 mutant)-β-lactamase-41G 197E | DHPETLVKVKDAEDQLGSGVDIAAFDPDKDGTIDLKEALAAGSAAFDKLDPDKDGTLDAKELKGRVSEADLKKLDPSNSGTLDKKEYLAAVEAQFKAANPDNDGTIDARELASPAGSALVNLIRGSGARVGYIELDLNSGKILESFRPEERFPMMSTFKVLLCGAVLSRVDAGQEQLGRRIHYSQNDLVEYSPVTEKHLTDGMTVRELCSAAITMSDNTAANLLLTTIGGPKELTAFLHNMGDHVTRLDRWEPELNEAIPNDERDTTMPVAMATTLRKLLTGGSGVDIAAFDPDKDGTIDLKEALAAGSAAFDKLDPDKDGTLDAKELKGRVSEADLKKLDPSNSGTLDKKEYLAAVEAQFKAANPDNDGTIDARELASPAGSALVNLIRGSGLLTLASRQQLIDWMEADKVAGPLLRSALPAGWFIADKSGAGERGSRGIIAALGPDGKPSRIVVIYTTGSQATMDERNRQIAEIGASLIKHWGKLAAALEHHHHHH |
| 2Lanmodulin(EF3 mutant)-β-lactamase-41G 197E thermostable variant | DHPETLVKVKDAEDQLGSGVDIAAFDPDKDGTIDLKEALAAGSAAFDKLDPDKDGTLDAKELKGRVSEADLKKLDPSNSGTLDKKEYLAAVEAQFKAANPDNDGTIDARELASPAGSALVNLIRGSGARVGYIELDLNSGQILESFRPEERFPMMSTFKVLLCGAVLARVDAGQEQLDRRIHYSQNDLVEYSPVTEKHLTDGMTVRELCSAAITMSDNTAANLLLKTIGGPKELTAFLRNMGDHVTRLDRWEPELNEAIPNDERDTTMPAAMATTLRKLLTGGSGVDIAAFDPDKDGTIDLKEALAAGSAAFDKLDPDKDGTLDAKELKGRVSEADLKKLDPSNSGTLDKKEYLAAVEAQFKAANPDNDGTIDARELASPAGSALVNLIRGSGLLTLASRQQLIDWMEADKVAGPLLRSALPAGWFIADKSGAGERGSRGIIAALGPDGKPSRIVVIYTTGSQATMDERNRQIAEIGASLIKHWGKLAAALEHHHHHH |
| 2Lanmodulin(EF1,3 mutant)-β-lactamase-41G 197E | DHPETLVKVKDAEDQLGSGVDIAAFDPSKSGTIDLKEALAAGSAAFDKLDPDKDGTLDAKELKGRVSEADLKKLDPSNSGTLDKKEYLAAVEAQFKAANPDNDGTIDARELASPAGSALVNLIRGSGARVGYIELDLNSGKILESFRPEERFPMMSTFKVLLCGAVLSRVDAGQEQLGRRIHYSQNDLVEYSPVTEKHLTDGMTVRELCSAAITMSDNTAANLLLTTIGGPKELTAFLHNMGDHVTRLDRWEPELNEAIPNDERDTTMPVAMATTLRKLLTGGSGVDIAAFDPSKSGTIDLKEALAAGSAAFDKLDPDKDGTLDAKELKGRVSEADLKKLDPSNSGTLDKKEYLAAVEAQFKAANPDNDGTIDARELASPAGSALVNLIRGSGLLTLASRQQLIDWMEADKVAGPLLRSALPAGWFIADKSGAG |

| | |
|---|---|
| | ERGSRGIIAALGPDGKPSRIVVIYTTGSQATMDERNRQIAEIGASLIKHWGKLAA<br>ALEHHHHHH |
| 2Lanmodulin(EF3,4 mutant)-β-lactamase-41G 197E | DHPETLVKVKDAEDQLGSGVDIAAFDPDKDGTIDLKEALAAGSAAFDKLDPDKD<br>GTLDAKELKGRVSEADLKKLDPSNSGTLDKKEYLAAVEAQFKAANPSNSGTIDAR<br>ELASPAGSALVNLIRGSGARVGYIELDLNSGKILESFRPEERFPMMSTFKVLLCGA<br>VLSRVDAGQEQLGRRIHYSQNDLVEYSPVTEKHLTDGMTVRELCSAAITMSDNT<br>AANLLLTTIGGPKELTAFLHNMGDHVTRLDRWEPELNEAIPNDERDTTMPVAM<br>ATTLRKLLTGGSGVDIAAFDPDKDGTIDLKEALAAGSAAFDKLDPDKDGTLDAKE<br>LKGRVSEADLKKLDPSNSGTLDKKEYLAAVEAQFKAANPSNSGTIDARELASPAG<br>SALVNLIRGSGLLTLASRQQLIDWMEADKVAGPLLRSALPAGWFIADKSGAGER<br>GSRGIIAALGPDGKPSRIVVIYTTGSQATMDERNRQIAEIGASLIKHWGKLAAALE<br>HHHHHH |
| 2Lanmodulin(EF2,3, 4 mutant)-β-lactamase-41G 197E | DHPETLVKVKDAEDQLGSGVDIAAFDPDKDGTIDLKEALAAGSAAFDKLDPSKS<br>GTLDAKELKGRVSEADLKKLDPSNSGTLDKKEYLAAVEAQFKAANPSNSGTIDAR<br>ELASPAGSALVNLIRGSGARVGYIELDLNSGKILESFRPEERFPMMSTFKVLLCGA<br>VLSRVDAGQEQLGRRIHYSQNDLVEYSPVTEKHLTDGMTVRELCSAAITMSDNT<br>AANLLLTTIGGPKELTAFLHNMGDHVTRLDRWEPELNEAIPNDERDTTMPVAM<br>ATTLRKLLTGGSGVDIAAFDPDKDGTIDLKEALAAGSAAFDKLDPSKSGTLDAKEL<br>KGRVSEADLKKLDPSNSGTLDKKEYLAAVEAQFKAANPSNSGTIDARELASPAGS<br>ALVNLIRGSGLLTLASRQQLIDWMEADKVAGPLLRSALPAGWFIADKSGAGERG<br>SRGIIAALGPDGKPSRIVVIYTTGSQATMDERNRQIAEIGASLIKHWGKLAAALEH<br>HHHHH |
| Lanmodulin(EF3 mutant)-404-GDH | DVPLIPSQFAKAKSENFDKKVILSNLNKPHALLWGPDNQIWLTERATGKILRVNP<br>ESGSVKTVFQVPEIVNDADGQNGLLGFAFHPDFKNNPYIYISGTFKNPKSTDKEL<br>PNQTIIRRYTYNKSTDTLEKPVDLLAGLPSSKDHQSGRLVIGPDQKIYYTIGDQGR<br>NQLAYLFLPNQAQHTPTQQELNGKDYHTYMGKVLRLNLDGSIPKDNPSFNGVV<br>SHIYTLGHRNPQGLAFTPNGKLLQSEQGPNSDDEINLIVKGGNYGWPNVAGYK<br>DDSGYAYANYSAAANKTIKDLAQNGVKVAAGVPVTKESEWTGKNFVPPLKTLY<br>TVQDTYNYNDPTCGEMTYICWPTVAPSSAYVYKGGKKAITGWENTLLVPSLKR<br>GVIFRIKLDPTYSTTYDDAVPMFKSGSGVDIAAFDPDKDGTIDLKEALAAGSAAF<br>DKLDPDKDGTLDAKELKGRVSEADLKKLDPSNSGTLDKKEYLAAVEAQFKAANP<br>DNDGTIDARELASPAGSALVNLIRGSGNRYRDVIASPDGNVLYVLTDTAGNVQK<br>DDGSVTNTLENPGSLIKFTYKAKKLAAALEHHHHHH |
| modified pACYCDuet Lanmodulin-β-lactamase-253G | GGCACTGATGAGGGTGTCAGTGAAGTGCTTCATGTGGCAGGAGAAAAAAG<br>GCTGCACCGGTGCGTCAGCAGAATATGTGATACAGGATATATTCCGCTTCCT<br>CGCTCACTGACTCGCTACGCTCGGTCGTTCGACTGCGGCGAGCGGAAATGG<br>CTTACGAACGGGGCGGAGATTTCCTGGAAGATGCCAGGAAGATACTTAACA<br>GGGAAGTGAGAGGGCCGCGGCAAAGCCGTTTTTCCATAGGCTCCGCCCCCC<br>TGACAAGCATCACGAAATCTGACGCTCAAATCAGTGGTGGCGAAACCCGAC<br>AGGACTATAAAGATACCAGGCGTTTCCCCCTGGCGGCTCCCTCGTGCGCTCTC<br>CTGTTCCTGCCTTTCGGTTTACCGGTGTCATTCCGCTGTTATGGCCGCGTTTGT<br>CTCATTCCACGCCTGACACTCAGTTCCGGGTAGGCAGTTCGCTCCAAGCTGG<br>ACTGTATGCACGAACCCCCCGTTCAGTCCGACCGCTGCGCCTTATCCGGTAAC<br>TATCGTCTTGAGTCCAACCCGGAAAGACATGCAAAAGCACCACTGGCAGCA<br>GCCACTGGTAATTGATTTAGAGGAGTTAGTCTTGAAGTCATGCGCCGGTTAA<br>GGCTAAACTGAAAGGACAAGTTTTGGTGACTGCGCTCCTCCAAGCCAGTTAC<br>CTCGGTTCAAAGAGTTGGTAGCTCAGAGAACCTTCGAAAAACCGCCCTGCAA<br>GGCGGTTTTTTCGTTTTCAGAGCAAGAGATTACGCGCAGACCAAAACGATCT<br>CAAGAAGATCATCTTATTAATCAGATAAAATATTTCTAGATTTCAGTGCAATT<br>TATCTCTTCAAATGTAGCACCTGAAGTCAGCCCCATACGATATAAGTTGTAAT |

| | |
|---|---|
| | TCTCATGTTAGTCATGCCCCGCGCCCACCGGAAGGAGCTGACTGGGTTGAAG<br>GCTCTCAAGGGCATCGGTCGAGATCCCGGTGCCTAATGAGTGAGCTAACTTA<br>CATTAATTGCGTTGCGCTCACTGCCCGCTTTCCAGTCGGGAAACCTGTCGTGC<br>CAGCTGCATTAATGAATCGGCCAACGCGCGGGGAGAGGCGGTTTGCGTATT<br>GGGCGCCAGGGTGGTTTTTCTTTTCACCAGTGAGACGGGCAACAGCTGATTG<br>CCCTTCACCGCCTGGCCCTGAGAGAGTTGCAGCAAGCGGTCCACGCTGGTTT<br>GCCCCAGCAGGCGAAAATCCTGTTTGATGGTGGTTAACGAAGATCCTTTGAT<br>CTTTTCTACGGGGTCTGACGCTCAGTGGAACGAAAACTCACGTTAAGGGATT<br>TTGGTCATGACCTATTTGTTTATTTTTCTAAATACATTCAAATATGTATCCGCT<br>CATGAGACAATAACCCTGATAAATGCTTCAATCATGATTGAAAAAGGAAGAG<br>TCCATGGATAAACATTTATTGGCTAAAATTGCTTTATTAGGCGCTGCTCAGCT<br>AGTTACACTCTCAGCATTTGCTGATCATCCGGAGACATTAGTAAAAGTAAAA<br>GACGCCGAAGATCAACTGGGGGCTCGGGTTGGCTACATTGAGCTTGATCTTA<br>ATAGCGGGAAGATACTTGAAAGTTTTCGTCCCGAGGAACGTTTTCCGATGAT<br>GTCAACTTTTAAGGTACTGCTTTGTGGTGCCGTTCTGAGCCGTGTAGACGCG<br>GGTCAAGAACAGTTGGGTAGAAGAATTCACTATTCACAGAATGACCTGGTG<br>GAATACTCCCCCGTTACGGAAAAGCATTTGACAGACGGGATGACGGTGCGG<br>GAACTGTGCTCAGCGGCGATTACTATGAGCGACAACACCGCCGCAAACTTGT<br>TGCTGACGACGATTGGTGGTCCTAAAGAATTAACGGCCTTTCTGCACAATAT<br>GGGGGATCACGTCACACGGCTGGATCGGTGGGAACCGGAGCTGAATGAAG<br>CTATACCTAACGATGAGAGAGACACCACAATGCCCGTGGCTATGGCGACTAC<br>ATTACGGAAGCTGTTGACAGGAGAGTTACTTACACTGGCGTCTAGACAGCA<br>GTTAATCGACTGGATGGAAGCTGATAAGGTAGCCGGGCCATTGTTACGGAG<br>CGCGTTACCTGCGGGGTGGTTTATCGCGGATAAGAGCGGAGCGGGGGAAC<br>GGGGTTCTAGAGGGATCATTGCTGCTTTGGGACCGGACGGTTCCGGTGTTG<br>ATATCGCCGCGTTCGATCCGGACAAAGATGGCACGATAGACTTAAAGGAAG<br>CTCTTGCTGCAGGCTCAGCAGCTTTTGACAAATTAGACCCCGACAAGGACGG<br>AACGCTTGACGCGAAAGAGCTGAAGGGTCGCGTATCCGAGGCCGACCTTAA<br>AAAGTTAGACCCCGATAATGACGGTACTTTGGATAAAAAAGAGTATTTGGCA<br>GCGGTTGAGGCACAGTTCAAAGCGGCAAACCCGGACAACGACGGGACCATC<br>GACGCCAGAGAGCTTGCCAGCCCAGCGGGTTCCGCCTTGGTAAACTTGATAA<br>GAGGCTCTGGCAAGCCGAGTCGCATCGTGGTAATATATACAACTGGTTCTCA<br>GGCCACAATGGATGAGCGGAATAGACAGATAGCCGAAATTGGCGCTAGCTT<br>AATAAAGCACTGGGGCAAGCTTGCGGCCGCATAATGCTTAAGTCGAACAGA<br>AAGTAATCGTATTGTACACGGCCGCATAATCGAAATAACTCACGTTAAGGGA<br>TTTTGGTCATGACCTATTTGTTTATTTTTCTAAATACATTCAAATATGTATCCG<br>CTCATGAGACAATAACCCTGATAAATGCTTCAATCATGATTGAAAAAGGAAG<br>AGTCATATGGACAAGCACTTGTTAGCAAAGATTGCATTATTGGGGGCAGCAC<br>AACTCGTAACTCTAAGTGCTTTCGCAGACAGATCTCAATTGGATATCGGCCG<br>GCCACGCGATCGCTGACGTCGGTACCGGAGGTAGTGGCTCCGGTGGTTCAG<br>GTGGCGGTAGCGGTAGCTCCGGAGGAAGTGGCGGATCTGGGGGTGGAAAG<br>CGTCGTTGGAAAAAGAACTTTATAGCCGTGAGTGCTGCCAATCGGTAGCTCG<br>AGCCTAGGCTGCTGCCACCGCTGAGCAATAACTAGCATAACCCCTTGGGGCC<br>TCTAAACGGGTCTTGAGGGGTTTTTTGCTGAAACCTCAGGCATTTGAGAAGC<br>ACACGGTCACACTGCTTCCGGTAGTCAATAAACCGGTAAACCAGCAATAGAC<br>ATAAGCGGCTATTTAACGACCCTGCCCTGAACCGACGACCGGGTCGAATTTG<br>CTTTCGAATTTCTGCCATTCATCCGCTTATTATCACTTATTCAGGCGTAGCACC<br>AGGCGTTTAAGGGCACCAATAACTGCCTTAAAAAAATTACGCCCCGCCCTGC<br>CACTCATCGCAGTACTGTTGTAATTCATTAAGCATTCTGCCGACATGGAAGCC<br>ATCACAGACGGCATGATGAACCTGAATCGCCAGCGGCATCAGCACCTTGTCG<br>CCTTGCGTATAATATTTGCCCATAGTGAAAACGGGGGCGAAGAAGTTGTCCA |

| | |
|---|---|
| | TATTGGCCACGTTTAAATCAAAACTGGTGAAACTCACCCAGGGATTGGCTGA<br>GACGAAAAACATATTCTCAATAAACCCTTTAGGGAAATAGGCCAGGTTTTCA<br>CCGTAACACGCCACATCTTGCGAATATATGTGTAGAAACTGCCGGAAATCGT<br>CGTGGTATTCACTCCAGAGCGATGAAAACGTTTCAGTTTGCTCATGGAAAAC<br>GGTGTAACAAGGGTGAACACTATCCCATATCACCAGCTCACCGTCTTTCATTG<br>CCATACGGAACTCCGGATGAGCATTCATCAGGCGGGCAAGAATGTGAATAA<br>AGGCCGGATAAAACTTGTGCTTATTTTTCTTTACGGTCTTTAAAAAGGCCGTA<br>ATATCCAGCTGAACGGTCTGGTTATAGGTACATTGAGCAACTGACTGAAATG<br>CCTCAAAATGTTCTTTACGATGCCATTGGGATATATCAACGGTGGTATATCCA<br>GTGATTTTTTTCTCCATTTTAGCTTCCTTAGCTCCTGAAAATCTCGATAACTCA<br>AAAAATACGCCCGGTAGTGATCTTATTTCATTATGGTGAAAGTTGGAACCTCT<br>TACGTGCCGATCAACGTCTCATTTTCGCCAAAAGTTGGCCCAGGGCTTCCCG<br>GTATCAACAGGGACACCAGGATTTATTTATTCTGCGAAGTGATCTTCCGTCAC<br>AGGTATTTATTCGGCGCAAAGTGCGTCGGGTGATGCTGCCAACTTACTGATT<br>TAGTGTATGATGGTGTTTTTGAGGTGCTCCAGTGGCTTCTGTTTCTATCAGCT<br>GTCCCTCCTGTTCAGCTACTGACGGGGTGGTGCGTAACGGCAAAAGCACCGC<br>CGGACATCAGCGCTAGCGGAGTGTATACTGGCTTACTATGTT |
| modified pACYCDuet 2Lanmodulin-β-lactamase-41G,197E | GGCACTGATGAGGGTGTCAGTGAAGTGCTTCATGTGGCAGGAGAAAAAAG<br>GCTGCACCGGTGCGTCAGCAGAATATGTGATACAGGATATATTCCGCTTCCT<br>CGCTCACTGACTCGCTACGCTCGGTCGTTCGACTGCGGCGAGCGGAAATGG<br>CTTACGAACGGGGCGGAGATTTCCTGGAAGATGCCAGGAAGATACTTAACA<br>GGGAAGTGAGAGGGCCGCGGCAAAGCCGTTTTTCCATAGGCTCCGCCCCC<br>TGACAAGCATCACGAAATCTGACGCTCAAATCAGTGGTGGCGAAACCCGAC<br>AGGACTATAAAGATACCAGGCGTTTCCCCTGGCGGCTCCCTCGTGCGCTCTC<br>CTGTTCCTGCCTTTCGGTTTACCGGTGTCATTCCGCTGTTATGGCCGCGTTTGT<br>CTCATTCCACGCCTGACACTCAGTTCCGGGTAGGCAGTTCGCTCCAAGCTGG<br>ACTGTATGCACGAACCCCCCGTTCAGTCCGACCGCTGCGCCTTATCCGGTAAC<br>TATCGTCTTGAGTCCAACCCGGAAAGACATGCAAAAGCACCACTGGCAGCA<br>GCCACTGGTAATTGATTTAGAGGAGTTAGTCTTGAAGTCATGCGCCGGTTAA<br>GGCTAAACTGAAAGGACAAGTTTTGGTGACTGCGCTCCTCCAAGCCAGTTAC<br>CTCGGTTCAAAGAGTTGGTAGCTCAGAGAACCTTCGAAAAACCGCCCTGCAA<br>GGCGGTTTTTTCGTTTTCAGAGCAAGAGATTACGCGCAGACCAAAACGATCT<br>CAAGAAGATCATCTTATTAATCAGATAAAATATTTCTAGATTTCAGTGCAATT<br>TATCTCTTCAAATGTAGCACCTGAAGTCAGCCCCATACGATATAAGTTGTAAT<br>TCTCATGTTAGTCATGCCCCGCGCCCACCGGAAGGAGCTGACTGGGTTGAAG<br>GCTCTCAAGGGCATCGGTCGAGATCCCGGTGCCTAATGAGTGAGCTAACTTA<br>CATTAATTGCGTTGCGCTCACTGCCCGCTTTCCAGTCGGGAAACCTGTCGTGC<br>CAGCTGCATTAATGAATCGGCCAACGCGCGGGGAGAGGCGGTTTGCGTATT<br>GGGCGCCAGGGTGGTTTTTCTTTTCACCAGTGAGACGGGCAACAGCTGATTG<br>CCCTTCACCGCCTGGCCCTGAGAGAGTTGCAGCAAGCGGTCCACGCTGGTTT<br>GCCCCAGCAGGCGAAAATCCTGTTTGATGGTGGTTAACGAAGATCCTTTGAT<br>CTTTTCTACGGGGTCTGACGCTCAGTGGAACGAAAACTCACGTTAAGGGATT<br>TTGGTCATGACCTATTTGTTTATTTTTCTAAATACATTCAAATATGTATCCGCT<br>CATGAGACAATAACCCTGATAAATGCTTCAATCATGATTGAAAAAGGAAGAG<br>TCCATGGATAAACATTTATTGGCTAAAATTGCTTTATTAGGCGCTGCTCAGCT<br>AGTTACACTCTCAGCATTTGCTGATCATCCGGAGACATTAGTAAAAGTAAAA<br>GACGCCGAAGATCAACTGGGTTCCGGTGTTGATATCGCCGCGTTCGATCCGG<br>ACAAAGATGGCACGATAGACTTAAAGGAAGCTCTTGCTGCAGGCTCAGCAG<br>CTTTTGACAAATTAGACCCCGACAAGGACGGAACGCTTGACGCGAAAGAGC<br>TGAAGGGTCGCGTATCCGAGGCCGACCTTAAAAAGTTAGACCCCGATAATG<br>ACGGTACTTTGGATAAAAAAGAGTATTTGGCAGCGGTTGAGGCACAGTTCA |

| | |
|---|---|
| | AAGCGGCAAACCCGGACAACGACGGGACCATCGACGCCAGAGAGCTTGCCA GCCCAGCGGGTTCCGCCTTGGTAAACTTGATAAGAGGCTCTGGCGCTCGGG TTGGCTACATTGAGCTTGATCTTAATAGCGGGAAGATACTTGAAAGTTTTCG TCCCGAGGAACGTTTTCCGATGATGTCAACTTTTAAGGTACTGCTTTGTGGTG CCGTTCTGAGCCGTGTAGACGCGGGTCAAGAACAGTTGGGTAGAAGAATTC ACTATTCACAGAATGACCTGGTGGAATACTCCCCCGTTACGGAAAAGCATTT GACAGACGGGATGACGGTGCGGGAACTGTGCTCAGCGGCGATTACTATGAG CGACAACACCGCCGCAAACTTGTTGCTGACGACGATTGGTGGTCCTAAAGAA TTAACGGCCTTTCTGCACAATATGGGGGATCACGTCACACGGCTGGATCGGT GGGAACCGGAGCTGAATGAAGCTATACCTAACGATGAGAGAGACACCACAA TGCCCGTGGCTATGGCGACTACATTACGGAAGCTGTTGACAGGAGGTTCCG GTGTTGATATCGCCGCGTTCGATCCGGACAAAGATGGCACGATAGACTTAAA GGAAGCTCTTGCTGCAGGCTCAGCAGCTTTTGACAAATTAGACCCCGACAAG GACGGAACGCTTGACGCGAAAGAGCTGAAGGGTCGCGTATCCGAGGCCGA CCTTAAAAAGTTAGACCCCGATAATGACGGTACTTTGGATAAAAAAGAGTAT TTGGCAGCGGTTGAGGCACAGTTCAAAGCGGCAAACCCGGACAACGACGG GACCATCGACGCCAGAGAGCTTGCCAGCCCAGCGGGTTCCGCCTTGGTAAA CTTGATAAGAGGCTCTGGCTTACTTACACTGGCGTCTAGACAGCAGTTAATC GACTGGATGGAAGCTGATAAGGTAGCCGGGCCATTGTTACGGAGCGCGTTA CCTGCGGGGTGGTTTATCGCGGATAAGAGCGGAGCGGGGGAACGGGGTTC TAGAGGGATCATTGCTGCTTTGGGACCGGACGGAAAGCCGAGTCGCATCGT GGTAATATATACAACTGGTTCTCAGGCCACAATGGATGAGCGGAATAGACA GATAGCCGAAATTGGCGCTAGCTTAATAAAGCACTGGGGCAAGCTTGCGGC CGCATAATGCTTAAGTCGAACAGAAAGTAATCGTATTGTACACGGCCGCATA ATCGAAATAACTCACGTTAAGGGATTTTGGTCATGACCTATTTGTTTATTTTTC TAAATACATTCAAATATGTATCCGCTCATGAGACAATAACCCTGATAAATGCT TCAATCATGATTGAAAAAGGAAGAGTCATATGGACAAGCACTTGTTAGCAAA GATTGCATTATTGGGGGCAGCACAACTCGTAACTCTAAGTGCTTTCGCAGAC AGATCTCAATTGGATATCGGCCGGCCACGCGATCGCTGACGTCGGTACCGG AGGTAGTGGCTCCGGTGGTTCAGGTGGCGGTAGCGGTAGCTCCGGAGGAA GTGGCGGATCTGGGGGTGGAAAGCGTCGTTGGAAAAAGAACTTTATAGCCG TGAGTGCTGCCAATCGGTAGCTCGAGCCTAGGCTGCTGCCACCGCTGAGCAA TAACTAGCATAACCCCTTGGGGCCTCTAAACGGGTCTTGAGGGGTTTTTTGC TGAAACCTCAGGCATTTGAGAAGCACACGGTCACACTGCTTCCGGTAGTCAA TAAACCGGTAAACCAGCAATAGACATAAGCGGCTATTTAACGACCCTGCCCT GAACCGACGACCGGGTCGAATTTGCTTTCGAATTTCTGCCATTCATCCGCTTA TTATCACTTATTCAGGCGTAGCACCAGGCGTTTAAGGGCACCAATAACTGCC TTAAAAAAATTACGCCCCGCCCTGCCACTCATCGCAGTACTGTTGTAATTCAT TAAGCATTCTGCCGACATGGAAGCCATCACAGACGGCATGATGAACCTGAAT CGCCAGCGGCATCAGCACCTTGTCGCCTTGCGTATAATATTTGCCCATAGTGA AAACGGGGGCGAAGAAGTTGTCCATATTGGCCACGTTTAAATCAAAACTGG TGAAACTCACCCAGGGATTGGCTGAGACGAAAAACATATTCTCAATAAACCC TTTAGGGAAATAGGCCAGGTTTTCACCGTAACACGCCACATCTTGCGAATAT ATGTGTAGAAACTGCCGGAAATCGTCGTGGTATTCACTCCAGAGCGATGAAA ACGTTTCAGTTTGCTCATGGAAAACGGTGTAACAAGGGTGAACACTATCCCA TATCACCAGCTCACCGTCTTTCATTGCCATACGGAACTCCGGATGAGCATTCA TCAGGCGGGCAAGAATGTGAATAAAGGCCGGATAAAACTTGTGCTTATTTTT CTTTACGGTCTTTAAAAAGGCCGTAATATCCAGCTGAACGGTCTGGTTATAG GTACATTGAGCAACTGACTGAAATGCCTCAAAATGTTCTTTACGATGCCATTG GGATATATCAACGGTGGTATATCCAGTGATTTTTTTCTCCATTTTAGCTTCCTT AGCTCCTGAAAATCTCGATAACTCAAAAAATACGCCCGGTAGTGATCTTATTT |

| | |
|---|---|
| | CATTATGGTGAAAGTTGGAACCTCTTACGTGCCGATCAACGTCTCATTTTCGC<br>CAAAAGTTGGCCCAGGGCTTCCCGGTATCAACAGGGACACCAGGATTTATTT<br>ATTCTGCGAAGTGATCTTCCGTCACAGGTATTTATTCGGCGCAAAGTGCGTC<br>GGGTGATGCTGCCAACTTACTGATTTAGTGTATGATGGTGTTTTTGAGGTGC<br>TCCAGTGGCTTCTGTTTCTATCAGCTGTCCCTCCTGTTCAGCTACTGACGGGG<br>TGGTGCGTAACGGCAAAAGCACCGCCGGACATCAGCGCTAGCGGAGTGTAT<br>ACTGGCTTACTATGTT |
| modified pColaDuet<br>-β-lactamase<br><br>(coloured<br>NcoI/HIndIII) | ACAAAACGCCACTGGCAGCAGCCATTGGTAACTGAGAATTAGTGGATTTAG<br>ATATCGAGAGTCTTGAAGTGGTGGCCTAACAGAGGCTACACTGAAAGGACA<br>GTATTTGGTATCTGCGCTCCACTAAAGCCAGTTACCAGGTTAAGCAGTTCCCC<br>AACTGACTTAACCTTCGATCAAACCGCCTCCCCAGGCGGTTTTTTCGTTTACA<br>GAGCAGGAGATTACGACGATCGTAAAAGGATCTCAAGAAGATCCTTTACGG<br>ATTCCCGACACCATCACTCTAGATTTCAGTGCAATTTATCTCTTCAAATGTAGC<br>ACCTGAAGTCAGCCCCATACGATATAAGTTGTAATTCTCATGTTAGTCATGCC<br>CCGCGCCCACCGGAAGGAGCTGACTGGGTTGAAGGCTCTCAAGGGCATCGG<br>TCGAGATCCCGGTGCCTAATGAGTGAGCTAACTTACATTAATTGCGTTGCGC<br>TCACTGCCCGCTTTCCAGTCGGGAAACCTGTCGTGCCAGCTGCATTAATGAAT<br>CGGCCAACGCGCGGGGAGAGGCGGTTTGCGTATTGGGCGCCAGGGTGGTT<br>TTTCTTTTCACCAGTGAGACGGGCAACAGCTGATTGCCCTTCACCGCCTGGCC<br>CTGAGAGAGTTGCAGCAAGCGGTCCACGCTGGTTTGCCCCAGCAGGCGAAA<br>ATCCTGTTTGATGGTGGTTAACGGCGGGATATAACATGAGCTGTCTTCGGTA<br>TCGTCGTATCCCACTACCGAGATGTCCGCACCAACGCGCAGCCCGGACTCGG<br>TAATGGCGCGCATTGCGCCCAGCGCCATCTGATCGTTGGCAACCAGCATCGC<br>AGTGGGAACGATGCCCTCATTCAGCATTTGCATGGTTTGTTGAAAACCGGAC<br>ATGGCACTCCAGTCGCCTTCCCGTTCCGCTATCGGCTGAATTTGATTGCGAGT<br>GAGATATTTATGCCAGCCAGCCAGACGCAGACGCGCCGAGACAGAACTTAA<br>TGGGCCCGCTAACAGCGCGATTTGCTGGTGACCCAATGCGACCAGATGCTCC<br>ACGCCCAGTCGCGTACCGTCTTCATGGGAGAAAATAATACTGTTGATGGGTG<br>TCTGGTCAGAGACATCAAGAAATAACGCCGGAACATTAGTGCAGGCAGCTT<br>CCACAGCAATGGCATCCTGGTCATCCAGCGGATAGTTAATGATCAGCCCACT<br>GACGCGTTGCGCGAGAAGATTGTGCACCGCCGCTTTACAGGCTTCGACGCC<br>GCTTCGTTCTACCATCGACACCACCACGCTGGCACCCAGTTGATCGGCGCGA<br>GATTTAATCGCCGCGACAATTTGCGACGGCGCGTGCAGGGCCAGACTGGAG<br>GTGGCAACGCCAATCAGCAACGACTGTTTGCCCGCCAGTTGTTGTGCCACGC<br>GGTTGGGAATGTAATTCAGCTCCGCCATCGCCGCTTCCACTTTTTCCCGCGTT<br>TTCGCAGAAACGTGGCTGGCCTGGTTCACCACGCGGGAAACGGTCTGATAA<br>GAGACACCGGCATACTCTGCGACATCGTATAACGTTACTGGTTTCACATTCAC<br>CACCCTGAATTGACTCTCTTCCGGGCGCTATCATGCCATACCGCGAAAGGTTT<br>TGCGCCATTCGATGGTGTCCGGGATCTCGACGCTCTCCCTTATGCGACTCCTG<br>CATTAGGAAAGCCGCATAATGCttaagttgacaattaatcatcggctcgtataatgtgtGG<br>AATTGTGAGCGGATAACAATTCCCCTCTAGAAATAATTTTGTTTAACTTTAAG<br>AAGGAGATATACCATGGATAAACATTTATTGGCTAAAATTGCTTTATTAGGC<br>GCTGCTCAGCTAGTTACACTCTCAGCATTTGCTGATCATCCGGAGACATTAGT<br>AAAAGTAAAAGACGCCGAAGATCAACTGGGGGCTCGGGTTGGCTACATTGA<br>GCTTGATCTTAATAGCGGGAAGATACTTGAAAGTTTTCGTCCCGAGGAACGT<br>TTTCCGATGATGTCAACTTTTAAGGTACTGCTTTGTGGTGCCGTTCTGAGCCG<br>TGTAGACGCGGGTCAAGAACAGTTGGGTAGAAGAATTCACTATTCACAGAA<br>TGACCTGGTGGAATACTCCCCCGTTACGGAAAAGCATTTGACAGACGGGAT<br>GACGGTGCGGGAACTGTGCTCAGCGGCGATTACTATGAGCGACAACACCGC<br>CGCAAACTTGTTGCTGACGACGATTGGTGGTCCTAAAGAATTAACGGCCTTT<br>CTGCACAATATGGGGGATCACGTCACACGGCTGGATCGGTGGGAACCGGAG |

| | |
|---|---|
| | CTGAATGAAGCTATACCTAACGATGAGAGAGACACCACAATGCCCGTGGCT<br>ATGGCGACTACATTACGGAAGCTGTTGACAGGAGAGTTACTTACACTGGCGT<br>CTAGACAGCAGTTAATCGACTGGATGGAAGCTGATAAGGTAGCCGGGCCAT<br>TGTTACGGAGCGCGTTACCTGCGGGGTGGTTTATCGCGGATAAGAGCGGAG<br>CGGGGGAACGGGGTTCTAGAGGGATCATTGCTGCTTTGGGACCGGACGGA<br>AAGCCGAGTCGCATCGTGGTAATATATACAACTGGTTCTCAGGCCACAATGG<br>ATGAGCGGAATAGACAGATAGCCGAAATTGGCGCTAGCTTAATAAAGCACT<br>GGGGCAAGCTTGCGGCCGCACTCGAGCACCACCACCACCACCACTGAGATC<br>CGGCTGCTAACAAAGCCCGAAAGGAAGCTGAGTTGGCTGCTGCCACCGCTG<br>aGATCTCAATTGGATATCGGCCGGCCACGCGATCGCTGACGTCGGTACCCTC<br>GAGTCTGGTAAAGAAACCGCTGCTGCGAAATTTGAACGCCAGCACATGGAC<br>TCGTCTACTAGCGCAGCTTAATTAACCTAGGCTGCTGCCACCGCTGAGCAAT<br>AACTAGCATAACCCCTTGGGGCCTCTAAACGGGTCTTGAGGGGTTTTTTGCT<br>GAAACCTCAGGCATTTGAGAAGCACACGGTCACACTGCTTCCGGTAGTCAAT<br>AAACCGGTAAACCAGCAATAGACATAAGCGGCTATTTAACGACCCTGCCCTG<br>AACCGACGACAAGCTGACGACCGGGTCTCCGCAAGTGGCACTTTTCGGGGA<br>AATGTGCGCGGAACCCCTATTTGTTTATTTTTCTAAATACATTCAAATATGTAT<br>CCGCTCATGAATTAATTCTTAGAAAAACTCATCGAGCATCAAATGAAACTGC<br>AATTTATTCATATCAGGATTATCAATACCATATTTTTGAAAAAGCCGTTTCTGT<br>AATGAAGGAGAAAACTCACCGAGGCAGTTCCATAGGATGGCAAGATCCTGG<br>TATCGGTCTGCGATTCCGACTCGTCCAACATCAATACAACCTATTAATTTCCCC<br>TCGTCAAAAATAAGGTTATCAAGTGAGAAATCACCATGAGTGACGACTGAAT<br>CCGGTGAGAATGGCAAAAGTTTATGCATTTCTTTCCAGACTTGTTCAACAGG<br>CCAGCCATTACGCTCGTCATCAAAATCACTCGCATCAACCAAACCGTTATTCA<br>TTCGTGATTGCGCCTGAGCGAGACGAAATACGCGGTCGCTGTTAAAAGGAC<br>AATTACAAACAGGAATCGAATGCAACCGGCGCAGGAACACTGCCAGCGCAT<br>CAACAATATTTTCACCTGAATCAGGATATTCTTCTAATACCTGGAATGCTGTTT<br>TCCCGGGGATCGCAGTGGTGAGTAACCATGCATCATCAGGAGTACGGATAA<br>AATGCTTGATGGTCGGAAGAGGCATAAATTCCGTCAGCCAGTTTAGTCTGAC<br>CATCTCATCTGTAACATCATTGGCAACGCTACCTTTGCCATGTTTCAGAAACA<br>ACTCTGGCGCATCGGGCTTCCCATACAATCGATAGATTGTCGCACCTGATTGC<br>CCGACATTATCGCGAGCCCATTTATACCCATATAAATCAGCATCCATGTTGGA<br>ATTTAATCGCGGCCTAGAGCAAGACGTTTCCCGTTGAATATGGCTCATACTCT<br>TCCTTTTTCAATATTATTGAAGCATTTATCAGGGTTATTGTCTCATGAGCGGA<br>TACATATTTGAATGTATTTAGAAAAATAAACAAATAGGCATGCTAGCGCAGA<br>AACGTCCTAGAAGATGCCAGGAGGATACTTAGCAGAGAGACAATAAGGCCG<br>GAGCGAAGCCGTTTTTCCATAGGCTCCGCCCCCCTGACGAACATCACGAAAT<br>CTGACGCTCAAATCAGTGGTGGCGAAACCCGACAGGACTATAAAGATACCA<br>GGCGTTTCCCCCTGATGGCTCCCTCTTGCGCTCTCCTGTTCCCGTCCTGCGGC<br>GTCCGTGTTGTGGTGGAGGCTTTACCCAAATCACCACGTCCCGTTCCGTGTA<br>GACAGTTCGCTCCAAGCTGGGCTGTGTGCAAGAACCCCCCGTTCAGCCCGAC<br>TGCTGCGCCTTATCCGGTAACTATCATCTTGAGTCCAACCCGGAAAGACACG |
| modified pColaDuet Lanmodulin(EF1 mutant)-β-lactamase-253G:<br><br>insert between NcoI/HindIII in modified pColaDuet -β-lactamase | catggataaacatttattggctaaaattgctttattaggcgctgctcagctagttacactctcagcattt<br>gctgatcatccggagacattagtaaaagtaaaagacgccgaagatcaactgggggctcgggttggc<br>tacattgagcttgatcttaatagcgggaagatacttgaaagttttcgtcccgaggaacgttttccgatg<br>atgtcaacttttaaggtactgctttgtggtgccgttctgagccgtgtagacgcgggtcaagaacagttg<br>ggtagaagaattcactattcacagaatgacctggtggaatactcccccgttacggaaaagcatttga<br>cagacgggatgacggtgcgggaactgtgctcagcggcgattactatgagcgacaacaccgccgcaa<br>acttgttgctgacgacgattggtggtcctaaagaattaacggcctttctgcacaatatgggggatcac<br>gtcacacggctggatcggtgggaaccggagctgaatgaagctatacctaacgatgagagagacacc<br>acaatgcccgtggctatggcgactacattacggaagctgttgacaggagagttacttacactggcgtc |

| | |
|---|---|
| | tagacagcagttaatcgactggatggaagctgataaggtagccgggccattgttacggagcgcgtta<br>cctgcggggtggtttatcgcggataagagcggagcgggggaacggggttctagagggatcattgctg<br>ctttgggaccggacggttccggtgttgatatcgccgcgttcgatccgagcaaaagtggcacgatagac<br>ttaaaggaagctcttgctgcaggctcagcagcttttgacaaattagaccccgacaaggacggaacgc<br>ttgacgcgaaagagctgaagggtcgcgtatccgaggccgaccttaaaaagttagaccccgataatg<br>acggtactttggataaaaaagagtatttggcagcggttgaggcacagttcaaagcggcaaacccgg<br>acaacgacgggaccatcgacgccagagagcttgccagcccagcgggttccgccttggtaaacttgat<br>aagaggctctggcaagccgagtcgcatcgtggtaatatatacaactggttctcaggccacaatggat<br>gagcggaatagacagatagccgaaattggcgctagcttaataaagcactggggca |
| modified pColaDuet Lanmodulin(EF2,3,4) -β-lactamase-253G:<br><br>insert between NcoI/HindIII in modified pColaDuet-β-lactamase | CATGGATAAACATTTATTGGCTAAAATTGCTTTATTAGGCGCTGCTCAGCTAG<br>TTACACTCTCAGCATTTGCTGATCATCCGGAGACATTAGTAAAAGTAAAAGA<br>CGCCGAAGATCAACTGGGGGCTCGGGTTGGCTACATTGAGCTTGATCTTAAT<br>AGCGGGAAGATACTTGAAAGTTTTCGTCCCGAGGAACGTTTTCCGATGATGT<br>CAACTTTTAAGGTACTGCTTTGTGGTGCCGTTCTGAGCCGTGTAGACGCGGG<br>TCAAGAACAGTTGGGTAGAAGAATTCACTATTCACAGAATGACCTGGTGGA<br>ATACTCCCCCGTTACGGAAAAGCATTTGACAGACGGGATGACGGTGCGGGA<br>ACTGTGCTCAGCGGCGATTACTATGAGCGACAACACCGCCGCAAACTTGTTG<br>CTGACGACGATTGGTGGTCCTAAAGAATTAACGGCCTTTCTGCACAATATGG<br>GGGATCACGTCACACGGCTGGATCGGTGGGAACCGGAGCTGAATGAAGCTA<br>TACCTAACGATGAGAGAGACACCACAATGCCCGTGGCTATGGCGACTACATT<br>ACGGAAGCTGTTGACAGGAGAGTTACTTACACTGGCGTCTAGACAGCAGTT<br>AATCGACTGGATGGAAGCTGATAAGGTAGCCGGGCCATTGTTACGGAGCGC<br>GTTACCTGCGGGGTGGTTTATCGCGGATAAGAGCGGAGCGGGGGAACGGG<br>GTTCTAGAGGGATCATTGCTGCTTTGGGACCGGACGGTTCCGGTGTTGATAT<br>CGCCGCGTTCGATCCGGACAAAGATGGCACGATAGACTTAAAGGAAGCTCT<br>TGCTGCAGGCTCAGCAGCTTTTGACAAATTAGACCCCAGCAAGAGCGGAAC<br>GCTTGACGCGAAAGAGCTGAAGGGTCGCGTATCCGAGGCCGACCTTAAAAA<br>GTTAGACCCCAGTAATAGCGGTACTTTGGATAAAAAAGAGTATTTGGCAGCG<br>GTTGAGGCACAGTTCAAAGCGGCAAACCCGAGCAACAGCGGGACCATCGAC<br>GCCAGAGAGCTTGCCAGCCCAGCGGGTTCCGCCTTGGTAAACTTGATAAGA<br>GGCTCTGGCAAGCCGAGTCGCATCGTGGTAATATATACAACTGGTTCTCAGG<br>CCACAATGGATGAGCGGAATAGACAGATAGCCGAAATTGGCGCTAGCTTAA<br>TAAAGCACTGGGGCA |
| modified pColaDuet Lanmodulin(EF2,3,4 mutant)-β-lactamase-41G:<br><br>insert between NcoI/HindIII in modified pColaDuet-β-lactamase | CATGGATAAACATTTATTGGCTAAAATTGCTTTATTAGGCGCTGCTCAGCTAG<br>TTACACTCTCAGCATTTGCTGATCATCCGGAGACATTAGTAAAAGTAAAAGA<br>CGCCGAAGATCAACTGGGTTCCGGTGTTGATATCGCCGCGTTCGATCCGGAC<br>AAAGATGGCACGATAGACTTAAAGGAAGCTCTTGCTGCAGGCTCAGCAGCT<br>TTTGACAAATTAGACCCCAGCAAGAGCGGAACGCTTGACGCGAAAGAGCTG<br>AAGGGTCGCGTATCCGAGGCCGACCTTAAAAAGTTAGACCCCAGTAATAGC<br>GGTACTTTGGATAAAAAAGAGTATTTGGCAGCGGTTGAGGCACAGTTCAAA<br>GCGGCAAACCCGAGCAACAGCGGGACCATCGACGCCAGAGAGCTTGCCAGC<br>CCAGCGGGTTCCGCCTTGGTAAACTTGATAAGAGGCTCTGGCGCTCGGGTTG<br>GCTACATTGAGCTTGATCTTAATAGCGGGAAGATACTTGAAAGTTTTCGTCCC<br>GAGGAACGTTTTCCGATGATGTCAACTTTTAAGGTACTGCTTTGTGGTGCCG<br>TTCTGAGCCGTGTAGACGCGGGTCAAGAACAGTTGGGTAGAAGAATTCACT<br>ATTCACAGAATGACCTGGTGGAATACTCCCCCGTTACGGAAAAGCATTTGAC<br>AGACGGGATGACGGTGCGGGAACTGTGCTCAGCGGCGATTACTATGAGCGA<br>CAACACCGCCGCAAACTTGTTGCTGACGACGATTGGTGGTCCTAAAGAATTA<br>ACGGCCTTTCTGCACAATATGGGGGATCACGTCACACGGCTGGATCGGTGG<br>GAACCGGAGCTGAATGAAGCTATACCTAACGATGAGAGAGACACCACAATG<br>CCCGTGGCTATGGCGACTACATTACGGAAGCTGTTGACAGGAGAGTTACTTA |

| | |
|---|---|
| | CACTGGCGTCTAGACAGCAGTTAATCGACTGGATGGAAGCTGATAAGGTAG<br>CCGGGCCATTGTTACGGAGCGCGTTACCTGCGGGGTGGTTTATCGCGGATA<br>AGAGCGGAGCGGGGGAACGGGGTTCTAGAGGGATCATTGCTGCTTTGGGA<br>CCGGACGGAAAGCCGAGTCGCATCGTGGTAATATATACAACTGGTTCTCAG<br>GCCACAATGGATGAGCGGAATAGACAGATAGCCGAAATTGGCGCTAGCTTA<br>ATAAAGCACTGGGGCA |
| modified pColaDuet Lanmodulin-β-lactamase-253G:<br><br>insert between NcoI/HindIII in modified pColaDuet-β-lactamase | CATGGATAAACATTTATTGGCTAAAATTGCTTTATTAGGCGCTGCTCAGCTAG<br>TTACACTCTCAGCATTTGCTGATCATCCGGAGACATTAGTAAAAGTAAAAGA<br>CGCCGAAGATCAACTGGGGGCTCGGGTTGGCTACATTGAGCTTGATCTTAAT<br>AGCGGGAAGATACTTGAAAGTTTTCGTCCCGAGGAACGTTTTCCGATGATGT<br>CAACTTTTAAGGTACTGCTTTGTGGTGCCGTTCTGAGCCGTGTAGACGCGGG<br>TCAAGAACAGTTGGGTAGAAGAATTCACTATTCACAGAATGACCTGGTGGA<br>ATACTCCCCCGTTACGGAAAAGCATTTGACAGACGGGATGACGGTGCGGGA<br>ACTGTGCTCAGCGGCGATTACTATGAGCGACAACACCGCCGCAAACTTGTTG<br>CTGACGACGATTGGTGGTCCTAAAGAATTAACGGCCTTTCTGCACAATATGG<br>GGGATCACGTCACACGGCTGGATCGGTGGGAACCGGAGCTGAATGAAGCTA<br>TACCTAACGATGAGAGAGACACCACAATGCCCGTGGCTATGGCGACTACATT<br>ACGGAAGCTGTTGACAGGAGAGTTACTTACACTGGCGTCTAGACAGCAGTT<br>AATCGACTGGATGGAAGCTGATAAGGTAGCCGGGCCATTGTTACGGAGCGC<br>GTTACCTGCGGGGTGGTTTATCGCGGATAAGAGCGGAGCGGGGGAACGGG<br>GTTCTAGAGGGATCATTGCTGCTTTGGGACCGGACGGTTCCGGTGTTGATAT<br>CGCCGCGTTCGATCCGGACAAAGATGGCACGATAGACTTAAAGGAAGCTCT<br>TGCTGCAGGCTCAGCAGCTTTTGACAAATTAGACCCCGACAAGGACGGAAC<br>GCTTGACGCGAAAGAGCTGAAGGGTCGCGTATCCGAGGCCGACCTTAAAAA<br>GTTAGACCCCGATAATGACGGTACTTTGGATAAAAAAGAGTATTTGGCAGCG<br>GTTGAGGCACAGTTCAAAGCGGCAAACCCGGACAACGACGGGACCATCGAC<br>GCCAGAGAGCTTGCCAGCCCAGCGGGTTCCGCCTTGGTAAACTTGATAAGA<br>GGCTCTGGCAAGCCGAGTCGCATCGTGGTAATATATACAACTGGTTCTCAGG<br>CCACAATGGATGAGCGGAATAGACAGATAGCCGAAATTGGCGCTAGCTTAA<br>TAAAGCACTGGGGCA |
| modified pColaDuet Lanmodulin(EF3 mutant)-β-lactamase-253G:<br><br>insert between NcoI/HindIII in modified pColaDuet-β-lactamase | CATGGATAAACATTTATTGGCTAAAATTGCTTTATTAGGCGCTGCTCAGCTAG<br>TTACACTCTCAGCATTTGCTGATCATCCGGAGACATTAGTAAAAGTAAAAGA<br>CGCCGAAGATCAACTGGGGGCTCGGGTTGGCTACATTGAGCTTGATCTTAAT<br>AGCGGGAAGATACTTGAAAGTTTTCGTCCCGAGGAACGTTTTCCGATGATGT<br>CAACTTTTAAGGTACTGCTTTGTGGTGCCGTTCTGAGCCGTGTAGACGCGGG<br>TCAAGAACAGTTGGGTAGAAGAATTCACTATTCACAGAATGACCTGGTGGA<br>ATACTCCCCCGTTACGGAAAAGCATTTGACAGACGGGATGACGGTGCGGGA<br>ACTGTGCTCAGCGGCGATTACTATGAGCGACAACACCGCCGCAAACTTGTTG<br>CTGACGACGATTGGTGGTCCTAAAGAATTAACGGCCTTTCTGCACAATATGG<br>GGGATCACGTCACACGGCTGGATCGGTGGGAACCGGAGCTGAATGAAGCTA<br>TACCTAACGATGAGAGAGACACCACAATGCCCGTGGCTATGGCGACTACATT<br>ACGGAAGCTGTTGACAGGAGAGTTACTTACACTGGCGTCTAGACAGCAGTT<br>AATCGACTGGATGGAAGCTGATAAGGTAGCCGGGCCATTGTTACGGAGCGC<br>GTTACCTGCGGGGTGGTTTATCGCGGATAAGAGCGGAGCGGGGGAACGGG<br>GTTCTAGAGGGATCATTGCTGCTTTGGGACCGGACGGTTCCGGTGTTGATAT<br>CGCCGCGTTCGATCCGGACAAAGATGGCACGATAGACTTAAAGGAAGCTCT<br>TGCTGCAGGCTCAGCAGCTTTTGACAAATTAGACCCCGACAAGGACGGAAC<br>GCTTGACGCGAAAGAGCTGAAGGGTCGCGTATCCGAGGCCGACCTTAAAAA<br>GTTAGACCCCAGTAATAGCGGTACTTTGGATAAAAAAGAGTATTTGGCAGCG<br>GTTGAGGCACAGTTCAAAGCGGCAAACCCGGACAACGACGGGACCATCGAC<br>GCCAGAGAGCTTGCCAGCCCAGCGGGTTCCGCCTTGGTAAACTTGATAAGA |

| | |
|---|---|
| | GGCTCTGGCAAGCCGAGTCGCATCGTGGTAATATATACAACTGGTTCTCAGGCCACAATGGATGAGCGGAATAGACAGATAGCCGAAATTGGCGCTAGCTTAATAAAGCACTGGGGCA |
